# Genome-Wide Identification of BAHD Superfamily and Functional Characterization of Bornyl Acetyltransferases Involved in the Bornyl Acetate Biosynthesis in *Wurfbainia villosa*

**DOI:** 10.3389/fpls.2022.860152

**Published:** 2022-03-28

**Authors:** Huilin Liang, Xiaojing Lin, Peng Yang, Yewen Sun, Qingwen Wu, Shamukaer Alimujiang, Haiying Zhao, Dongming Ma, Ruoting Zhan, Jinfen Yang

**Affiliations:** ^1^Key Laboratory of Chinese Medicinal Resource from Lingnan (Ministry of Education), Guangzhou University of Chinese Medicine, Guangzhou, China; ^2^School of Pharmaceutical Science, Guangzhou University of Chinese Medicine, Guangzhou, China; ^3^Hunan Provincial Key Laboratory for Synthetic Biology of Traditional Chinese Medicine, School of Pharmaceutical Sciences, Hunan University of Medicine, Huaihua, China

**Keywords:** bornyl acetate, borneol acetyltransferase, *Wurfbainia villosa*, BAHD gene family, alcohol acetyltransferase

## Abstract

Bornyl acetate (BA) is known as a natural aromatic monoterpene ester with a wide range of pharmacological and biological activities. Borneol acetyltransferase (BAT), catalyzing borneol and acetyl-CoA to synthesize BA, is alcohol acetyltransferase, which belongs to the BAHD super acyltransferase family, however, BAT, responsible for the biosynthesis of BA, has not yet been characterized. The seeds of *Wurfbainia villosa* (homotypic synonym: *Amomum villosum*) are rich in BA. Here we identified 64 members of the BAHD gene family from the genome of *W. villosa* using both PF02458 (transferase) and PF07247 (AATase) as Hidden Markov Model (HMM) to screen the BAHD genes. A total of sixty-four WvBAHDs are distributed on 14 chromosomes and nine unanchored contigs, clustering into six clades; three WvBAHDs with PF07247 have formed a separated and novel clade: clade VI. Twelve candidate genes belonging to clade I-a, I-b, and VI were selected to clone and characterize *in vitro*, among which eight genes have been identified to encode BATs acetylating at least one type of borneol to synthesize BA. All eight WvBATs can utilize (−)-borneol as substrates, but only five WvBATs can catalyze (+)-borneol, which is the endogenous borneol substrate in the seeds of *W. villosa*; WvBAT3 and WvBAT4 present the better catalytic efficiency on (+)-borneol than the others. The temporal and spatial expression patterns of *WvBATs* indicate that *WvBAT3* and *WvBAT4* are seed-specific expression genes, and their expression levels are correlated with the accumulation of BA, suggesting WvBAT3 and WvBAT4 might be the two key BATs for BA synthesis in the seeds of *W. villosa*. This is the first report on BAT responsible for the last biosynthetic step of BA, which will contribute to further studies on BA biosynthesis and metabolism engineering of BA in other plants or heterologous hosts.

## Introduction

Natural products have been a prominent source of pharmacologically active molecules in medicines for years, with advantages of effectiveness and low occurrence of side effects. Bornyl acetate (BA) has been reported for its rich pharmacological effects. BA has shown a high lipoxygenase inhibition, leading to the reduction of the inflammatory/allergic response, tumoral and Alzheimer’s disorders, and reducing 5-fluorouracil-induced intestinal mucositis (Zhang et al., 2017; Cutillas et al., 2018). BA has also been demonstrated to be the potential proliferation inhibitor against human MCF-7, HT-29, and H-1299 cells (Sajjadi et al., 2015). Moreover, it has been reported that BA has analgesic, antioxidant, whitening, anticancer, antitumor, antiabortion, and anti-anxiety effects, and it has antibacterial, insecticidal, and anesthetic effects symbiotically with other aromatic compounds in the volatile oil (Wang et al., 2011; Asghari et al., 2012; Ohtsubo et al., 2015; Perestrelo et al., 2016; Zhang et al., 2017; Ao et al., 2019). However, BA is only distributed in a few families of plants, such as *Zingiberaceae, Pinaceae*, *Cupressaceae*, *Rutaceae*, *Umbelliferae*, *Lamiaceae*, and *Anacardiaceae*. *Wurfbainia villosa* (homotypic synonym: *Amomum villosum*) is rich in BA; BA content in the seeds essential oil is 10.53% (50.35% of the total monoterpene content), higher than the contents of BA in other plants, such as *Laurus nobilis* (fruits, 4.40%), *Illicium pachyphyllum* (fruits, 4.01%), and *Valeriana Jatamansi* (root, 0.6–1.5%) (Liu et al., 2012; Raina and Negi, 2015; Wang et al., 2018; Fidan et al., 2019; Chen et al., 2020b). Therefore, *W. villosa* is a significant material for illuminating the BA biosynthetic pathway. *W. villosa* is a plant of the ginger family, and its fragrant dried fruit is known as Fructus Amomi (Chinese medicine name: Sharen). It is a well-known traditional Chinese medicine and is widely used in the treatment of digestive system diseases and daily cooking in China, especially in southern China. BA is the medicinal substance and quality index of *W. villosa*, and its content determines the quality of *W. villosa* (Commission of Chinese Materia Medica, 1999; Commission of Chinese Pharmacopoeia, 2015). In addition to high levels of BA, *W. villosa* contains a variety of terpene acetates with lower or trace levels, such as isobornyl acetate (IBA), nerolidyl acetate, and santalyl acetate, which are the unique aroma and flavor components of *W. villosa* relating to its pharmacological activities (Zhao et al., 2021b; Supplementary Table 1).

**FIGURE 1:**
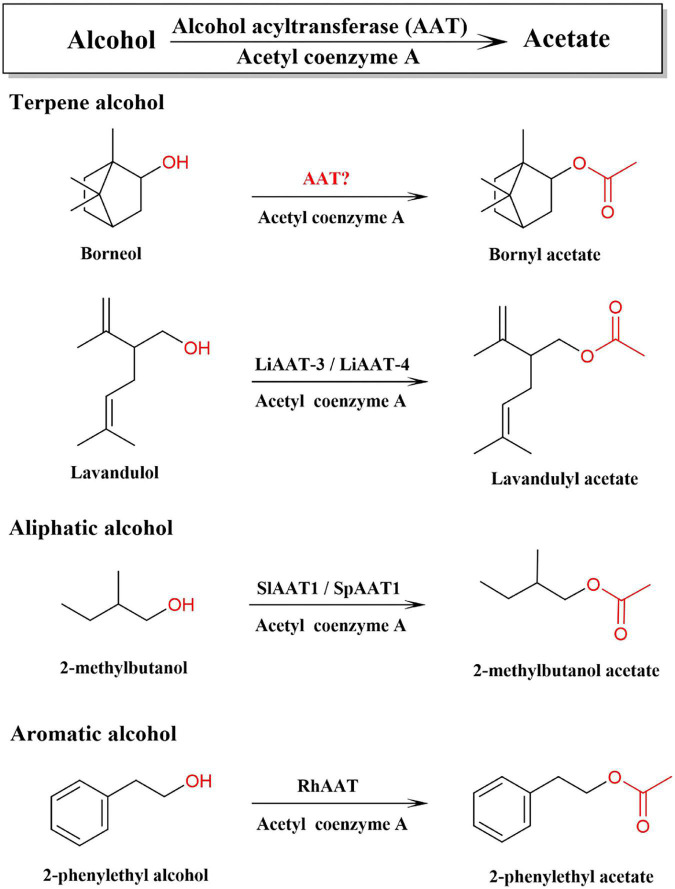
Alcohol acetyltransferase (AATs) involved in the biosynthetic pathway of acetate with different precursor alcohols.

In plants, all terpenoid skeletons are generated by the (MVA) pathway and the 2-C-methyl-D-erythritol-4-phosphate (MEP) pathway, which have been well documented (Vranová et al., 2013). Bornyl diphosphate synthase (BPPS) is the first key enzyme in the downstream pathway of BA biosynthesis, which catalyzes the monoterpene precursor, geranyl diphosphate (GPP), to generate bornyl diphosphate, the precursor of borneol. *BPPS* has been cloned and characterized from a few plants, such as WvBPPS from *W. villosa*, CbBPPS from *Cinnamomum burmannii* (Wang et al., 2018; Ma et al., 2021). Diphosphate diphosphatase is converted to borneol by dephosphorylation, and borneol is acetylated by alcohol acetyltransferase to synthesize BA. Alcohol acetyltransferase (AAT, EC 2.3.1.84) can catalyze terpene alcohol, aromatic alcohol, or aliphatic alcohol to synthesize aromatic volatile acetate in plants (Figure 1; Beekwilder et al., 2004; Souleyre et al., 2005). *AATs* have been cloned and identified from the fruits of strawberry, banana, apple, peach, and flowers of rose and lavender, and traditional herbs, such as *Celastrus angulatus*, *Ocimum basilicum*, and other plants (Supplementary Table 2; Beekwilder et al., 2004; Souleyre et al., 2005; Zhang et al., 2010; Sarker and Mahmoud, 2015; Dhar et al., 2020; Yan et al., 2020). BA is synthesized by acetylation of borneol, which has three optical isomers, including levorotatory, dextrorotatory, and racemate (Drienovská et al., 2020; Chánique et al., 2021). In contrast, chemically synthesized borneol contains four different stereoisomers, (+)-borneol, (−)-borneol, (+)-isoborneol, and (−)-isoborneol (Khine et al., 2020). However, the AAT responsible for the synthesis of BA and other monoterpene acetates in *W. villosa* has not yet been characterized; furthermore, the gene encoding borneol acetyltransferase (BAT) catalyzing different types of borneol to synthesize BA has not yet been reported to date.

Alcohol acetyltransferase is one important member of the BAHD acyltransferase family, which was named according to the first alphabet of the first four biochemically characterized enzymes of this family (BEAT, AHCT, HCBT, and DAT) and features with two highly conserved motifs: “HXXXD” and “DFGWG” (St-Pierre and De Luca, 2000). The BAHD members identified to date are all monomeric enzymes with a molecular mass ranging from 48 to 55 kDa (D’Auria, 2006). AATs are mainly high expressed in plant tissues which accumulate significant amounts of volatile esters, such as glandular trichomes, flowers (petals/stigmas), or fruits (pulp/receptacle), and some are expressed in leaves or stems (Beekwilder et al., 2004; Souleyre et al., 2005; Sarker and Mahmoud, 2015; Dhar et al., 2020; Yan et al., 2020). AATs expressed in fruits are maturation-induced. The substrates including alcohol precursors and acyl-CoA donors of AAT are broad, and one AAT might be involved in the synthesis and accumulation of multiple esters in plants at the same time (Ma et al., 2005; Balbontín et al., 2010; Nimitkeatkai et al., 2011; Cumplido-Laso et al., 2012; Souleyre et al., 2014). AAT can utilize more kinds of alcohol substrates *in vitro* other than the endogenous substrates, however, the biosynthesis of ester compounds mainly depends on the available substrates in the plant. There might be multiple AATs in the seeds of *W. villosa*, which is the major organ enriched with BA and other monoterpene acetates.

In this study, as part of the ongoing efforts to complete the BA biosynthesis pathway in *W. villosa*, we identified 64 BAHD members from the *W. villosa* genome database and selected 12 WvAAT candidate genes to clone and characterize. Eight genes were identified by biochemical assay to encode for BAT, converting at least one type of borneol to BA, and two of them were further proved to be the key BATs responsible for the BA synthesis in the seeds of *W. villosa*.

## Materials and Methods

### Plant Material

*Wurfbainia villosa* is from Yangchun City, Guangdong Province, China. The leaves, rhizome, flowers, and fruits from healthy plants were collected and frozen at −80°C. The fruits at different developmental stages: 30-days after flowering (DAF), 45-DAF, 60-DAF, 75-DAF, and 90-DAF were separated into pericarp and seeds.

### Genome-Wide Identification of BAHD Superfamily

To identify putative BAHD genes in *W. villosa*, we used the Protein Basic Logical Alignment Search Tool (BLASTP) (*E*-value cutoff of 1e-5) to compare *Arabidopsis thaliana* and *Malus domestica* BAHD protein sequences (Liu et al., 2020) with the *W. villosa* genomic data (unpublished). The amino-acid sequences of *A. thaliana* and *M. domestica* were obtained from phytozome.^1^ Then, based on the hidden Markov model (HMM) of the BAHD domain (PF02458) obtained from the Pfam database,^2^ we also identified candidate WvBAHDs using Hmmsearch (3.2.1) (Potter et al., 2018). Then, we used the Web CD-Search Tool^3^ and Web SMART^4^ to confirm the domains of the candidate WvBAHDs identified by the two methods mentioned above, and then their HXXXD and DFGWG motifs were inspected. Furthermore, PF07247 (alcohol acetyltransferase, AATase) was used to screen AAT as the supplement of BAHD genes. Collinear blocks of the WvBAHD were identified using MCSanX and the results of circular plots were generated using Circos (Wang et al., 2012). The amino acid sequences alignments and the maximum-likelihood (ML) phylogenetic tree with bootstraps of 1,000 were obtained by using the MUSCLE and IQ-TREE of TBtools (Chen et al., 2020a), and the clade of clustering adapted from Liu et al. (2020).

### Selection of Candidate WvAAT Genes Involved in Bornyl Acetate Biosynthesis

The WvAAT candidate genes were selected directly by their clade clustered into and their transcripts per million (TPM) expression value (from unpublished RNA-seq data) combined with the upstream gene *WvBPPS* in different tissues and the fruits at different developmental stages. To predict the substrate preference of WvAAT, the multi-sequence alignments and phylogenetic analysis with the reported AATs were performed using the ML method with the tool MEGA X (Kumar et al., 2018). The information of the AATs from other plants used for the phylogenetic analysis was shown in Supplementary Table 2. Candidates were further analyzed for a multiple sequence comparison analysis of the nucleotide and amino acid sequences using the software Jalview (Waterhouse et al., 2009). The conserved and active motifs predictions were used by the online tool MEME^5^ (Bailey and Elkan, 1994); the protein structure, signal peptide, and transit peptide of the WvAAT candidates were predicted and analyzed by online platform TMHMM 2.0,^6^ SignalP,^7^ and ChloroP 1.1 Server^8^ (Emanuelsson et al., 1999; Krogh et al., 2001; Petersen et al., 2011). According to the results of the sequence alignment phylogenetic tree, the evolutionary tree was beautified by the online tool iTOL^9^ (Letunic and Bork, 2021).

### Amplification of Full-Length WvAAT Candidate Gene

The extraction method of total RNA and complementary DNA (cDNA) was the same as the previous reports (Wang et al., 2018). The coding regions of AAT candidate genes were amplified from *W. villosa* cDNA using the Primer STAR Max DNA Polymerase (Takara, China) with appropriate primers (Supplementary Table 3). The PCR conditions used were the following: 98°C, 1 min; 98°C 10 s, 50–60°C, 15 s, 72°C, 15 s, 30 cycles; 72°C, 5 min. The purified PCR products were then ligated into pLB (Tiangen, China) or 007 vs. (TSINGKE, China) cloning vector, which were consequently transformed into *E. coli* DH5α cells and sequencing.

### Prokaryotic Expression and Purification of WvAAT Recombinant Protein

The full-length ORFs of the candidate WvAATs were ligated into pET32a (+) expression vector, using the In-Fusion Cloning Kit (Takara, China), and then transformed into the *Escherichia coli* Rosetta (DE3). The primers were described in Supplementary Table 3. Cells were grown at 37°C until the OD_600_ reaches.4–0.6, and induced at 16°C with isopropyl-β-D-thiogalactopyranoside (IPTG) at a final concentration of 10 μM for 16–20 h in Luria–Bertani (LB) media supplemented with 50 μg/μL carbenicillin and 25 μg/μL chloramphenicol. The recombinant protein was purified using NI-NTA resin (Qiagen, Hilden, Germany). The purified protein was dialyzed on a PD-10 desalting column (GE Healthcare).

### Enzyme Assay and Product Analysis of WvAAT Candidates

The *in vitro* enzymatic reactions were performed at 250–350 μL total volume [20 mM sodium hydrogen phosphate buffer (pH 9.0), 10% glycerol] containing 20–80 μg purified protein, 0.2 mM acetyl-CoA, and 0.02 mM terpene alcohol substrate [(+)-borneol, (−)-borneol, isoborneol, geraniol, nerol, α-terpineol, (−)-menthol, linalool and nerolidol]. According to the previous reports on AAT enzymatic assays (Croteau and Hooper, 1978; Sarker and Mahmoud, 2015) and the results of our preliminary experiments, the optimum pH and temperature for WvAATs were determined to be 9.0 and 32°C, respectively. All reaction mixture was incubated at 32°C for 6 h, then immediately overlaid with 250 μL hexane. The mixture was then centrifuged at 12,000 rpm for 5 min to separate the phases. The hexane extraction was analyzed using Agilent 7890B Gas Chromatograph with 5977A inert Mass Selective Detector (Agilent, United States). Helium was used as the carrier gas (1 mL/min) and then separated on the HP-5MS column (30 m × 250 μm × 0.25 μm film thickness). The gas chromatography (GC) oven temperature was programmed at an initial temperature of 35°C for 2 min with an increase of 12°C/min to 300°C. The temperature was then kept at 300°C for 5 min. For chiral compounds, CycloSil-B column (30 m × 0.25 mm id, 0.25 mm film thickness) was used for separation, initially at 50°C for 2 min, and then at 5°C/min from 50 to 180°C, increase to 230°C at 10°C/min, and hold at 230°C for 2 min. NIST14/Wiley275 Mass Spectral Library was used for metabolite identification. Meanwhile, the standards were also utilized for further identification. Each WvAAT was tested in triplicate.

### Volatile Terpenoid Extraction and Analysis

Approximately, 0.1 g of seeds at different developmental stages were ground frizzed in liquid nitrogen and extracted with 1 mL hexane using an ultrasonic cleaner for 30 min, and then incubated at 40°C for 1 h. The samples were then centrifuged at 10,000 rpm for 15 min and the resulting supernatants were pipetted into novel 2 mL tubes. One milliliter of hexane extract was pipetted into 1 mL vial for GC- mass spectrometry (MS) analysis. The extraction was analyzed using Agilent 7890B Gas Chromatograph with 5977A inert Mass Selective Detector (Agilent, United States). Helium was used as the carrier gas (1 mL/min) and then separated on the CycloSil-B column (30 m × 0.25 mm id, 0.25 mm film thickness). The GC oven temperature was programmed at an initial temperature of 35°C for 2 min, and then at 5°C/min from 35to 200°C, increase to 230°C at 10°C/min, and hold at 230°C for 2 min. The temperature was then kept at 240°C for 5 min. NIST14/Wiley275 Mass Spectral Library was used for metabolite identification. The terpene compounds were identified by the mass spectral library. The predominant terpene acetates and their precursor terpene alcohols in this research were further identified using their authentic standards. There were three biological replicates and three technical replicates for each organ.

### Quantitative Real-Time PCR of WvBATs

Based on the correlation analysis between transcriptome and ester data of *W. villosa*, eight identified WvBATs were selected for quantitative real-time PCR (qRT-PCR). The primers for real-time PCR of all the WvBAT genes were designed manually (Fluorescence-specific primers shown in Supplementary Table 3). The quantitative PCR (qPCR) of WvBATs was performed using 2xTSINGKE^®^Master qRT-PCR Mix-SYBR (+ UDG, TSINGKE, China) in the CFX96 real-time PCR detection system (Bio-Rad, United States). The WvBAT transcript levels were monitored using the internal reference gene TUA, and calculated using the 2^–ΔΔCt^ method. There were three biological replicates and three technical replicates for all experiments.

### Analysis of the WvBATs Promoters

Preliminary element analysis was performed on the approximate area of the promoter. The 2,000 bp non-coding region upstream of the *WvBATs* coding region was extracted through TBtools (Chen et al., 2020a) and then analyzed on the online website PlantCare^10^ (Lescot et al., 2002) to find out whether the element was related to seed-specific expression. The classification of the *cis-*acting elements of the promoters of functional genes was referred to Abdullah et al. (2018).

## Results

### Genome-Wide Identification of WvBAHD Gene Family and WvAAT Candidates

**FIGURE 2:**
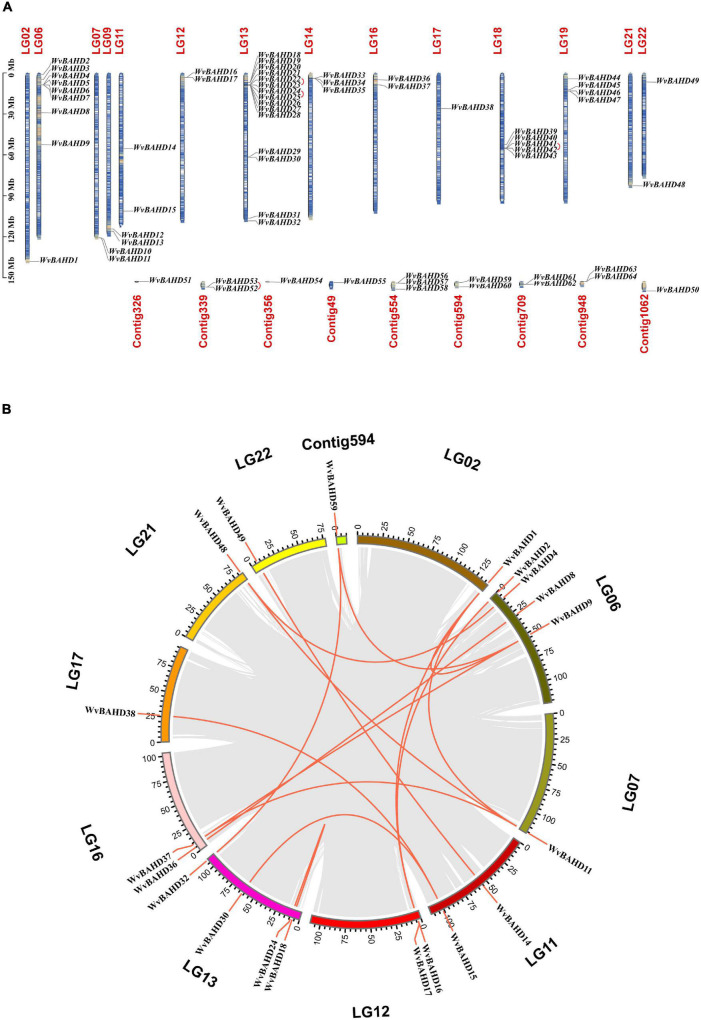
Gene location and collinearity analysis of the WvBAHD gene family. **(A)** Chromosome location and distribution analysis of WvBAHD genes. Tandem duplicated genes are linked by a red line. **(B)** Chromosomal duplication analysis of WvBAHD genes in circos graph. Red lines represent the syntenic gene pairs.

Sixty-one putative *BAHD* genes with complete ORF and two conserved motifs were identified from the genome database of *W. villosa* using the BAHD HMM configuration file (PF02458, transferase). In addition, three genes (*WvBAHD3*, *52*, and *53*) were screened out using PF07247 (AATase) as HMM. AATase (PF07247) comes from CL0149, a protein clan named CoA-acyltrans with a characteristic HXXXD motif, same as the transferase family (PF02458). The AATase family contains a number of AATs from bacteria and metazoa, catalyzing the esterification of isoamyl alcohol by acetyl-CoA, similar to the AATs from the plant (Minetoki et al., 1993; Zhu et al., 2015; Lin et al., 2016; Reyes-Sánchez et al., 2019). Accordingly, we speculated that these three genes screened by PF07247 (AATase) should be members of the *BAHD* superfamily. In total, 64 *WvBAHDs* have been identified as BAHD super acyltransferase family members (Supplementary Table 4), which are distributed on 14 chromosomes and nine unanchored contigs (Figure 2A). The genome synteny analysis showed that 15 segmental duplications (23.4%) and four tandem duplication (6.3%) events occurred, suggesting that segmental duplication events have an important contribution to the expansion of the *WvBAHD* gene family (Figure 2B). The prediction results of the *trans-*membrane function indicated that most *WvBAHDs* were located in the cytoplasm (Supplementary Table 5).

**FIGURE 3:**
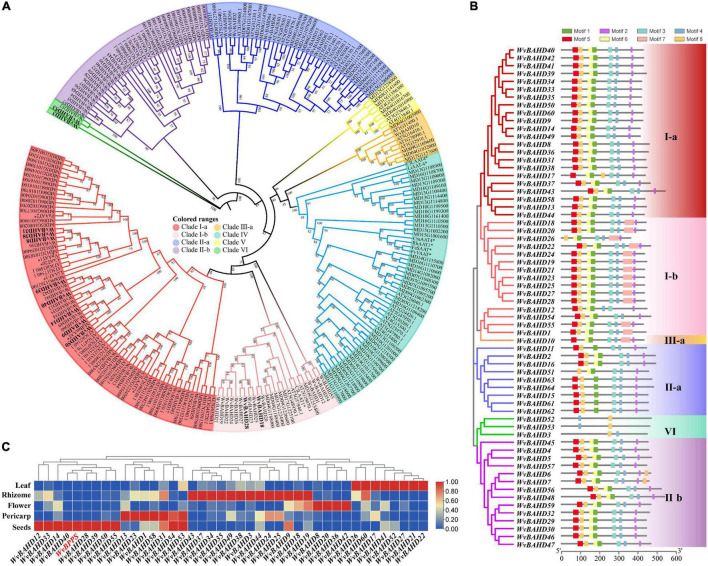
**(A)** Phylogenetic analysis of WvBAHDs from *Wurfbainia villosa*, AtBAHDs from *Arabidopsis thaliana*, and MdBAHDs from *Malus domestica*, and 14 identified AATs marked with asterisk. The phylogenetic tree was constructed by maximum-likelihood with 1,000 replications, and the clade of clustering was adapted from Liu et al. (2020). The WvAAT candidates were indicated in bold black font. **(B)** Conserved motifs analysis of WvBAHDs. Legend depicting the amino acid sequence of the conserved motifs were shown in Supplementary Figure 1. **(C)** Transcriptome differential expression analysis of *WvBAHDs* from clade om clare 1. **(C)** nd *WvBPPS*. Pericarp and seeds were from 60-DAF (Days after flowering) fruits.

**FIGURE 4:**
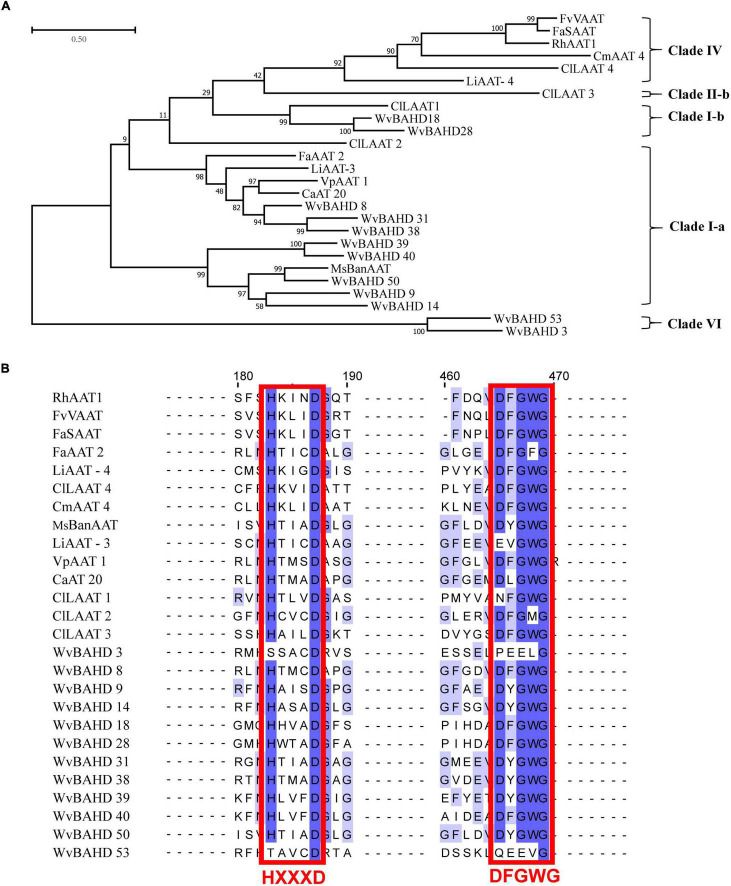
**(A)** Phylogenetic analysis of 12 WvAAT candidate genes and 14 identified AATs from other plants. The phylogenetic tree was constructed by maximum likelihood with 1,000 replications, and the clade of clustering was adapted from Liu et al. (2020). **(B)** Sequence comparison of HXXXD and DFGWG motifs in WvAATs and identified AATs proteins. The percentage identify threshold was 30% or above. Conserved motifs are highlighted in red. Supplementary Table 2 contains the GenBank accession numbers of each AAT sequence used.

To determine the classification of the *WvBAHD* gene family, we constructed a phylogenetic tree using amino acid sequences encoded by 235 *BAHD* genes from *W. villosa*, *A. thaliana*, and *M. domestica*, and 14 identified AATs from other plants. The 64 putative WvBAHDs were classified into five clades: I-a (twenty-two genes), I-b (fifteen genes), II-a (nine genes), II-b (fourteen genes), III-a (one gene), and VI (three genes) (Figure 3A). The genes belonging to clade I-a are involved in modifying aromatic and terpenoid alcohols in *A. thaliana* and *M. domestica*, therefore, we speculated that the WvBAHDs clustered into clade I-a in *W. villosa* might encode proteins with similar functions. However, the identified AAT were also clustered into clade I-b and clade IV, which is inconsistent with the reports that the members of clade I-b had functions related to the biosynthesis of lignin monomeric intermediates (Hoffmann et al., 2003; Petrik et al., 2014). This might be due to the phylogenetic tree algorithm and these enzymes have not been comprehensively identified to date. Furthermore, three AATase-WvBAHDs are clustered separately as a novel clade and named clade VI. Therefore, the range of WvAAT candidate genes we inferred here was expanded from clade I-a to clade I-b and clade VI. To confirm the correlation between the above clustering and conserved motifs, we detected eight conserved motifs in the WvBAHDsof *W. villosa* by MEME. Motif1 and Motif2 correspond to conserved motifs HXXXD and DFGWG, respectively (Figure 3B and Supplementary Figures 1, 2). The similarity in the type and distribution of conserved motifs in the same clade further supports the classification of evolutionary trees. Among the members of clade VI, the histidine residues from the HXXXD motif were substituted by threonine or serine, and four residues from the DFGWG motif were substituted, causing the clade VI members distant relation with other members of BAHD and other identified AATs (Figures 3A, 4).

Then, we compared the expression profiles of the genes from clade I and VI with *WvBPPS*, the key gene for borneol biosynthesis, and identified four genes (*WvBAHD14*, *WvBAHD40*, *WvBAHD39*, *WvBAHD50*; TPM >10) co-expressed with *WvBPPS* in 60-DAF seeds, suggesting that these genes might be the key genes involved in the borneol acetylation (Figure 3C). Furthermore, in clade I-a, WvBAHD8, WvBAHD38, and WvBAHD31 share high identity (>45%) with the reported AATs (CaAT20, LiAAT3, VpAAT1, and FaAAT2); WvBAHD9, WvBAHD14, WvBAHD39, WvBAHD40, and WvBAHD50 are clustered with MsBanAAT into a close branch; in clade I-b, WvBAHD18 and WvBAHD28 share approximately 40% identity with ClLAAT1, an AAT from *Citrus limon* (Figure 4). These reported AATs to show enzymatic affinity to geraniol and nerol *in vitro* (Aharoni et al., 2000; Supplementary Table 2), therefore, we speculated that these WvBAHDs might have AAT activity similar to these reported AATs. Thence, ten genes (*WvBAHD8*, *WvBAHD9*, *WvBAHD14*, *WvBAHD18*, *WvBAHD28*, *WvBAHD31*, *WvBAHD38*, *WvBAHD39*, *WvBAHD40*, and *WvBAHD50*) from clade I and two genes (*WvBAHD53* and *WvBAHD3*) from clade VI, which are all expressed in the seeds (Supplementary Table 6), were selected as candidate *WvAATs* for cloning and functional characterization.

### Eight WvAATs Were Characterized as Borneol Acetyltransferase

Enzymatic assays were conducted *in vitro* using recombinant proteins extracted and purified from *E. coli* expression strains (Supplementary Figure 3). The enzymatic assays showed that eight WvAAT recombinant proteins (WvBAHD8, WvBAHD14, WvBAHD28, WvBAHD39, WvBAHD40, WvBAHD50, WvBAHD3, and WvBAHD53) can acetylate geraniol and nerol, which are primary alcohols easily to be catalyzed, to form geranyl acetate and neryl acetate, respectively, even with the small amount of soluble proteins, verifying that these eight candidates have AAT enzymatic activity, and this method is effective for the AAT characterization *in vitro*, according to previous research (Shalit et al., 2003; Sarker and Mahmoud, 2015; Supplementary Figure 4). In the case of the other four candidate WvAATs, no geranyl acetate or neryl acetate was detected from the enzymatic reaction, and they could not catalyze bornyl-type substrate either.

**FIGURE 5:**
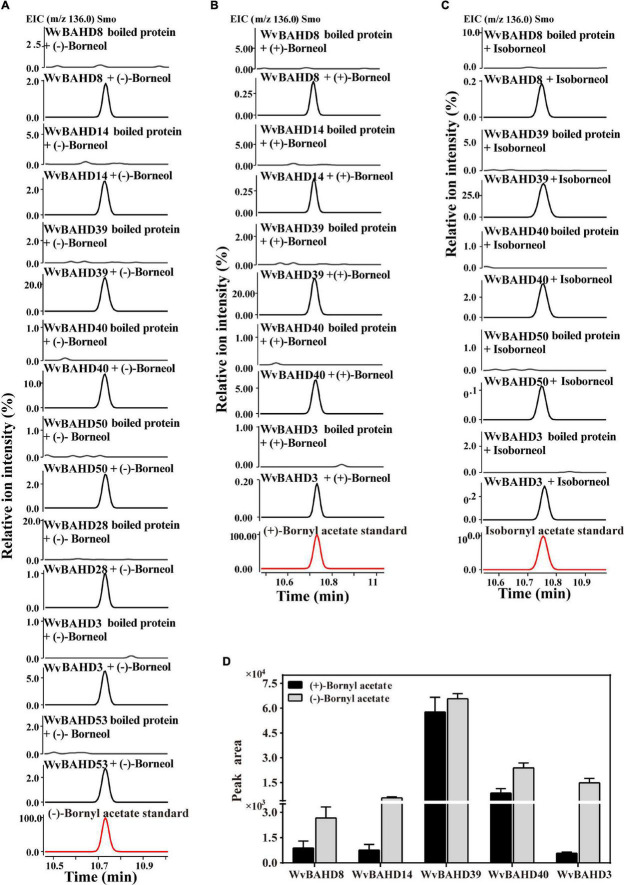
Functional characterization of WvBAHDs. **(A–C)** The gas chromatography (GC)–mass spectrometry (MS) chromatogram of the *in vitro* reaction products yielded by each WvBAHD using (−)-borneol, (+)-borneol, and isoborneol as the substrate, respectively. **(D)** The GC-MS chromatograms peak area (EIC, m/z 136.0) of products yielded by each WvBAHD at the same substrate concentration (enzyme concentrations vary among WvBAHDs), using (−)-borneol, (+)-borneol, and isoborneol as the substrate, respectively.

To determine the BAT activity of these eight WvAAT recombinant proteins mentioned above, the catalytic products using borneol-type substrates, including (−)-borneol, (+)-borneol, and isoborneol, were analyzed. The results reveal that all these WvAATs can catalyze the substrate (−)-borneol to produce (−)-BA, however, (+)-BA was only detected from the catalytic products of five WvAATs (WvBAHD8, WvBAHD14, WvBAHD39, WvBAHD40, and WvBAHD3) when (+)-borneol was used as substrate (Figures 5A,B); we thus concluded that these five WvAATs capable to catalyze both (−)-borneol and (+)-borneol have no strict substrate specificity for different optical isomers of borneol, while WvBAHD28, WvBAHD50, and WvBAHD53 have substrate specificity for (−)-borneol. Among these five WvAATs capable to catalyze both (−)-borneol and (+)-borneol, WvBAHD8, WvBAHD39, WvBAHD40, and WvBAHD3 can also catalyze isoborneol to produce IBA (Figure 5C). In addition to borneol-type substrate, more diverse substrates were used to perform the enzyme assay, and none of these WvAATs could react with tertiary alcohols, such as (−)-menthol, linalool, or nerolidol, to form relative acetate products, similar to that of LiAAT3 and LiAAT4 (Sarker and Mahmoud, 2015). Since all these WvAATs can convert at least one type of borneol to BA, demonstrating that they have the BAT activity, therefore they are renamed as WvBAT1–8, respectively (Table 1 and Supplementary Table 7). To compare the catalytic activity for different optical isomers of borneol substrate, the product peak areas of the five WvBATs which can catalyze both (−)-borneol and (+)-borneol, reacting with the same amount of each substrate, were analyzed. The results indicated that these WvBATs had the better catalytic ability for (−)-borneol than for (+)-borneol; notably, WvBAT3 (WvBAHD39) exhibited the best catalytic efficiency for (+)-borneol than the other WvBATs, followed by WvBAT4 (WvBAHD40) (Figure 5D). In addition, WvBAT3 is the only enzyme capable to acetylate the α-terpineol to synthesize α-terpinyl acetate (Table 1 and Supplementary Figure 5).

**TABLE 1:** The information of the characterized WvBATs in *Wurfbainia Villosa*.

Family in Pfam (HMMs)	BAHD family	Original ID	Renamed ID	Protein size (aa)	Protein molecular weight (kDa)	Catalytic substrate					
						(+)-Borneol	(−)-Borneol	Isoborneol	Geraniol	Nerol	α- Terpineol
Transferase (PF02458)	Clade I-a	WvBAHD8	WvBAT1	458	49.76	✓	✓	✓	✓	✓	n.d.
		WvBAHD14	WvBAT2	411	45.93	✓	✓	n.d.	✓	✓	n.d.
		WvBAHD39	WvBAT3	443	49.65	✓	✓	✓	✓	✓	✓
		WvBAHD40	WvBAT4	427	47.35	✓	✓	✓	✓	✓	n.d.
		WvBAHD50	WvBAT5	419	46.05	n.d.	✓	✓	✓	✓	n.d.
	Clade I-b	WvBAHD28	WvBAT6	435	47.21	n.d.	✓	n.d.	✓	✓	n.d.
AATase (PF07247)	Clade VI	WvBAHD3	WvBAT7	488	48.64	✓	✓	✓	✓	✓	n.d.
		WvBAHD53	WvBAT8	461	50.75	n.d.	✓	n.d.	✓	✓	n.d.

*“✓” indicated that the substrate can be converted to the corresponding acetate; “n.d.” indicated that no corresponding acetate was detected in the enzymatic reaction.*

### The Optical Configuration of Borneol and Bornyl Acetate in the Seeds of *Wurfbainia villosa*

**FIGURE 6:**
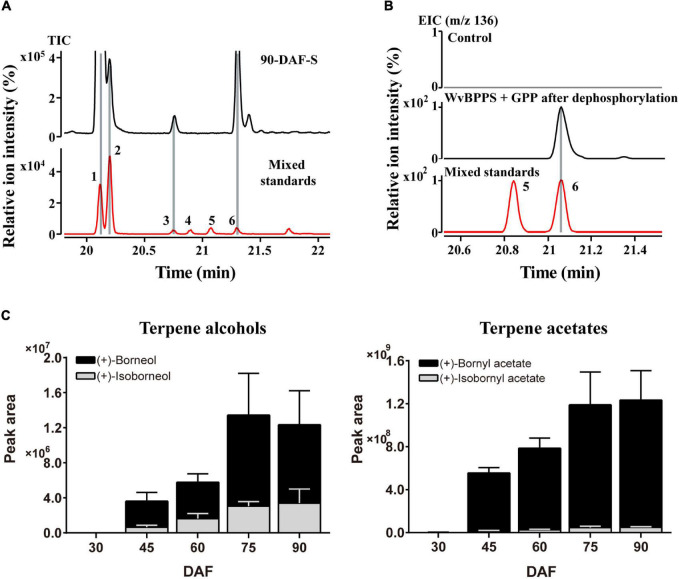
**(A)** The GC-MS chromatogram of the volatile terpene in the 90-DAF seeds of *Wurfbainia villosa*, and the mixed standards. **(B)** The GC-MS chromatogram of the products generated by WvBPPS protein after dephosphorylation and the mixed-borneol standard and its negative control was treated with boiled protein. The compounds of peaks: 1. bornyl acetate; 2. isobornyl acetate; 3. (+)-isoborneol; 4. (−)-isoborneol; 5. (−)-borneol; 6. (+)-borneol. **(C)** The GC-MS chromatographic peak (EIC, m/z 136.0) areas of (+)-borneol, (+)-isoborneol and their corresponding acetates (+)-bornyl acetate and (+)-isobornyl acetate, in seeds at different developmental stages of *W. villosa*.

As we mentioned above, most of the WvBATs present a better catalytic ability for (−)-borneol *in vitro*. (−)-Borneol and (+)-borneol are optical isomers and both of them are natural metabolites in plants, however, the optical configuration of endogenous borneol in the seeds of *W. villosa* and the WvBPPS-catalyzed product hasn’t been identified with the chiral column to date (Wang et al., 2018). Therefore, we used GC-MS with CycloSil-B column to identify the chiral configuration of borneol in the seeds of *W. villosa*, and that of the product in the enzymatic reaction of WvBPPS. The results showed that no (−)-borneol was detected but a significant amount of (+)-borneol and a small amount of isoborneol were detected in the mature (90-DAF) seeds of *W. villosa* (Figure 6A); this result was also observed in the volatile extract from seeds at different developmental stages (30-DAF to 75-DAF), and consistent with the observation that (+)-borneol was the WvBPPS-catalyzed product with phosphatase treatment (Figure 6B). Furthermore, the content of (+)-BA rises with the increasing of (+)-borneol throughout the seed development, peaking at 90 DAF; (+)-borneol has a substantially larger content than isoborneol, which matches the data that (+)-BA has a significantly higher amount than IBA (Figure 6C). These results suggest that (+)-borneol is the major endogenous borneol-type substrate in the seeds of *W. villosa*.

### Correlation Between Gene Expression Levels of WvBATs With Acetates Accumulation in *Wurfbainia villosa*

**FIGURE 7:**
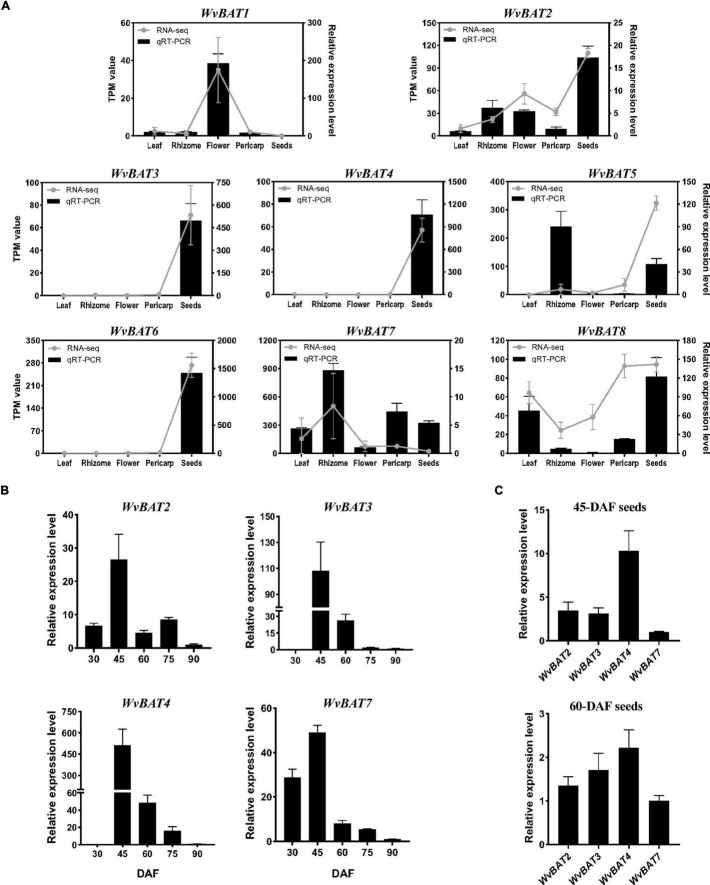
Expression pattern of *WvBATs*. **(A)** The relative expression levels of *WvBATs* in different tissues. Pericarp and seeds were from 60-days after flowering (DAF) fruits. Transcripts per million reads (TPM) value was from the RNA-sequencing (RNA-seq) data. Comparison of the expression levels of the *WvBATs*, capable to catalyze (+)-borneol, in seeds at different developmental stages **(B)** or at the same stage, 45-DAF or 60-DAF **(C)**. Data represent the means ± SDs (*n* = 3).

To analyze the temporal and spatial expression patterns of *WvBATs*, qRT-PCR was performed using the leaf, rhizome, flower, and 60-DAF fruit (separated into pericarp and seeds), and the seeds at five developmental stages. The result demonstrated that the relative expression levels of *WvBATs* were basically consistent with the TMP (transcripts per million) expression value from the RNA-sequencing (RNA-seq) data, except for *WvBAT5*; *WvBAT5* was actually expressed higher in rhizome than in the seeds. The expressional patterns of *WvBAT3*, *WvBAT4*, and *WvBAT6* are seed-specific, similar to that of *WvBPPS*. Although *WvBAT6* was expressed specifically in the seeds, the enzyme it encodes cannot catalyze (+)-borneol and isoborneol, indicating that it might participate in the biosynthesis of other acetates in the seeds. *WvBAT1* was expressed specifically in the flower, suggesting that it might be involved in the acetates biosynthesis in the flower. Furthermore, *WvBAT7* was expressed in all the organs with the highest level in the rhizome, indicating that it might be involved in the BA synthesis in the rhizome, which contains low levels of BA (Wang et al., 2018; Figure 7A).

As we mentioned above, (+)-borneol is the major endogenous substrate in the seeds, and BA accumulates in the seeds with an obvious occurrence at 45-DAF (Figure 6C); only four *WvBATs* (*WvBAT2*, *WvBAT3*, *WvBAT4*, and *WvBAT7*) are capable to catalyze (+)-borneol and expressed in the seeds as well. Therefore, to determine the major BAT(s) responsible for the BA biosynthesis, we investigated their expression patterns in the seeds at different developmental stages and compared their expression levels in 45-DAF and 60-DAF seeds. Of which, the expression levels of *WvBAT2* and *WvBAT7* peaked at 45 DAF, while their expression levels were much lower than *WvBAT4* (Figures 7B,C). Notably, both *WvBAT3* and *WvBAT4*, the seed-specific expressed genes, were not expressed at 30-DAF, but started to be highly expressed at the 45-DAF stage, similar to that the highest expression level of *WvBPPS* presented in 45-DAF, in accord with the initial accumulation levels of (+)-borneol and (+)-BA in the seeds (Wang et al., 2018; Figure 7B). In addition, *WvBAT4* was the highest-expressed gene in the seeds at 45-DAF and 60-DAF, and the expression level of *WvBAT3* in 60-DAF seeds is also higher than the other two *WvBATs* (Figure 7C). Considering WvBAT3 (WvBAHD39) is the enzyme presenting the best catalytic efficiency on (+)-borneol (Figure 5D), we speculated that both WvBAT3 and WvBAT4 are the key enzymes responsible for the synthesis of BA. In addition, IBA initial accumulation in the seeds also increased from 45-DAF (Figure 6C), implying that the WvBATs (WvBAT3, WvBAT4, and WvBAT7) enable to catalyze (+)-isoborneol, were also involved in the IBA synthesis in the seeds of *W. villosa*.

### Promoter Analysis of the WvBATs

**FIGURE 8:**
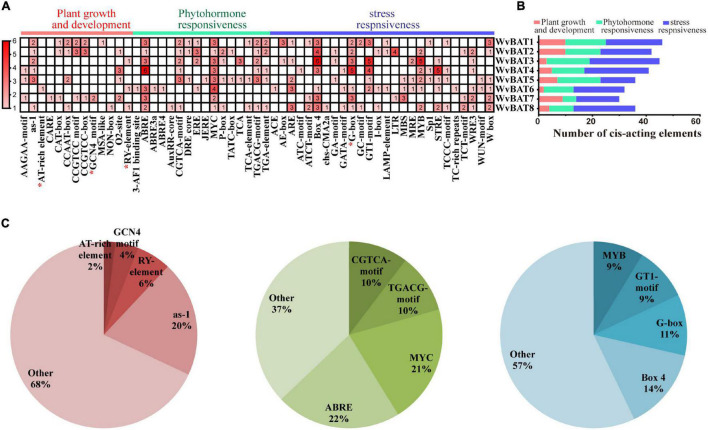
**(A)** The number of *cis-*acting elements in *WvBATs*, indicated by the intensity of the red color and numbers in the grid. The cis-acting elements with seed-special expression were indicated by a red asterisk. **(B)** Histograms indicate the number of *cis-*acting elements involved in plant growth and development, phytohormone responsiveness, or stress responsiveness. **(C)** Pie charts showing the ratio of different *cis-*acting elements in each structural category.

To ascertain the potential biological roles of WvBATs in *W. villosa*, the element analysis was performed on the approximate area of the promoter of *WvBAT1*–8 genes. In total, 308 *cis-*acting elements were identified, and they were grouped into three categories, phytohormone responsiveness, stress responsiveness, and plant growth and development (Figures 8A,B). The greatest proportion of *cis-*acting regulatory elements related to phytohormone response was ABRE elements that were associated with abscisic acid (ABA, 22%), followed by MYC (21%), TGACG-motif (10%), and CGTCA-motif (10%); the latter three elements were related to methyl jasmonate (MeJA). Nearly half of all the *cis-*acting elements were associated with stress responsiveness (161/304), of which the most common 4 *cis-*acting elements were the box 4 (14%), G-box (11%), GT1-motif (9%), and MYB (9%), the first three were associated with responsiveness to light, and the last one was related to water stress (Figure 8C). These results suggested that *WvBATs* might be induced or suppressed by MeJA and ABA, and involved in plant responses to a variety of abiotic stressors.

We previously observed that promoters of *WvBPPS* featured a seed-specific expression element GCN4-motif, which was consistent with the main accumulation of borneol in the seeds (Zhao et al., 2021a). The seed-expression *cis-*acting elements include the GCN4 motif involved in the endosperm *cis-*elements (Onodera et al., 2001), RY-element, and AT-rich element involved in seed-specific regulation (Bäumlein et al., 1992; Ellerström et al., 1996), and G-box (Izawa et al., 1993, 1994). Expectedly, excluding *WvBAT5*, these *WvBAT* promoters all contained *cis-*acting elements related to seed expression; for example, the *WvBAT7* promoter contained two GCN4 motifs, and the promoters of *WvBAT1*, *WvBAT2*, *WvBAT4*, *WvBAT6*, and *WvBAT7* contained G-box.

## Discussion

### A Novel Pfam Hidden Markov Model PF07247 for Identifying BAHD Acyltransferase Genes in Plant

There are relatively few studies devoted to the HMM of the BAHD super acyltransferase family for screening BAHD members in plants. PF02458, the HMM of the transferase family, which belong to CL0149 with a characteristic HXXXD motif, was obtained by comparing the HMM in the Pfam database of reported members of the BAHD family, which was consistent with the results of previous studies (Liu et al., 2020; Kumar et al., 2021). We identified 61 WvBAHDs by PF02458 from *W. villosa* genomic data, featured “HXXXD” and “DFGWG,” which is consistent with the characteristic of the BAHD family.

In our investigation, besides PF02458, the other Pfam HMM PF07247 of the AATase family, which belongs to CL0149 as well, was also used to identify the BAHD family members of *W. villosa*. Accordingly, three WvBAHDs (WvBAHD3, WvBAHD52, and WvBAHD53) annotated as AATase had been screened out. However, there was no reported HMM profile of BAHD members similar to PF07247. Actually, “AATase” usually describes the AAT of bacteria, metazoan, and fungi, such as yeast *Saccharomyces* and *Kluyveromyces* (Zhu et al., 2015; Reyes-Sánchez et al., 2019). To further confirm whether these three AATase-WvBAHDs belong to the BAHD acyltransferase family, we constructed another phylogenetic tree using 64 WvBAHDs and the identified BAHDs from other species (Supplementary Table 8) based on the cluster system from D’Auria, which clusters BAHD acyltransferases into six major groups (D’Auria, 2006; Sarker and Mahmoud, 2015). Unexpectedly, these three AATase-WvBAHDs were all clustered into the clade V, which consists mostly of AATs involved in volatile ester biosynthesis (Supplementary Figure 6); however, they are not clustered closely with the other identified AATs from plants, which might be due to amino acid residue substitutions in their DFGWG motif, one of the conserved motifs of BAHD acyltransferase family. Notably, WvBAHD3 and WvBAHD53 do have the activity of AAT, and both can acetylate borneol, geraniol, and nerol (Table 1). Therefore, this work has verified “PF07247/AATase” is feasible for screening BAHD superfamily members in the plant, thus, the BAHD family would be enriched by adding new AAT members.

### The First Report on the Borneol Acetyltransferases Responsible for the Last Biosynthetic Step of Bornyl Acetate

Bornyl acetate is the aromatic monoterpene ester with a wide range of pharmacological and biological activities, but only a few plants accumulate a significant amount of BA. BPPS, the enzyme catalyzing the synthesis of borneol precursor, the first step on BA downstream biosynthesis pathway of BA, has been identified from other plants, including *Salvia officinalis* (SoBPPS), *Lippia dulcis* (LdBPPS), *Lavandula angustifolia* (LaBPPS), and *Cinnamomum burmannii* (CbBPPS) (Wise et al., 1998; Despinasse et al., 2017; Hurd et al., 2017; Ma et al., 2021); except for *Lippia dulcis*, these plants all contain BA (Al-Dhubiab, 2012; Arceusz et al., 2013), however, the gene encoding BAT, catalyzing the acetylation of borneol, has not yet been reported. In this study, based on the identification of the BAHD family, we cloned and characterized eight *WvBATs* from *W. villosa*. This is the first report on the BAT responsible for the last biosynthetic step of BA. These WvBATs can acetylate at least one type of borneol to synthesize BA, and they are substrate-promiscuous enzymes, similar to other AATs. AAT is the substrate-promiscuous enzyme, which can acetylate different types of alcohols and acyl-CoA to synthesize aromatic volatile ester compounds in plants (Beekwilder et al., 2004; Souleyre et al., 2005). However, the type and content of acylation products depend on the presence and content of endogenous substrates in plants. For example, the type of acetate ester synthesized in mint depends not on the specificity of AATs, but on the availability of the terpene alcohol in mint oil (Croteau and Hooper, 1978). With regard to *W. villosa*, the volatile oil of the seeds contains high levels of (+)-borneol, much lower levels of isoborneol, and only trace levels of α-terpineol, accordingly, (+)-borneol is the predominant endogenous substrate for WvBATs in the seeds. Two WvBATs, WvBAT3 and WvBAT4, capable of acetylating (+)-borneol with higher efficiency, and expressed in the seeds with positive correlation to the BA accumulation, might be the key enzymes collaborate to synthesize BA in the seeds of *W. villosa*. This speculation is consistent with the previous reports that the plants contain several AATs which might collaborate in the synthesis of a single ester (Cumplido-Laso et al., 2012; Sarker and Mahmoud, 2015).

**FIGURE 9:**
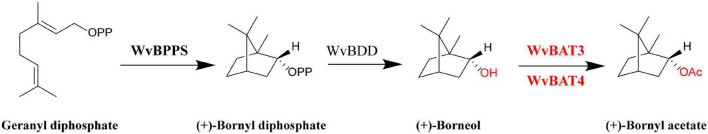
Biosynthetic pathway of bornyl acetate (BA) in *Wurfbainia villosa*. BPPS: bornyl diphosphate synthase; BDD, bornyl diphosphate diphosphatase; BAT, bornyl acyltransferase. The WvBAT in red was identified to be the key enzyme involved in the synthesis of bornyl acetate (BA) in seeds.

In summary, based on the biochemical characterization of WvBATs in this study and previous literature (Wise et al., 1998; Despinasse et al., 2017; Wang et al., 2018), we illuminated the downstream biosynthetic pathway of BA in *W. villosa* (Figure 9). This work will contribute to a better understanding of the biosynthesis of BA and other acetates in *W. villosa*, and lay the foundation for further studies on BA biosynthesis and metabolism engineering of BA in other plants or heterologous hosts.

## Data Availability Statement

The datasets presented in this study can be found in online repositories. The names of the repository/repositories and accession number(s) can be found in the article/Supplementary Material.

## Author Contributions

JY and HL designed the experiments and wrote the manuscript. HL, XL, PY, YS, QW, SA, and HZ performed the experiments and analyzed the data. RZ and DM revised the manuscript. All authors contributed to the article and approved the submitted version.

## Conflict of Interest

The authors declare that the research was conducted in the absence of any commercial or financial relationships that could be construed as a potential conflict of interest.

## Publisher’s Note

All claims expressed in this article are solely those of the authors and do not necessarily represent those of their affiliated organizations, or those of the publisher, the editors and the reviewers. Any product that may be evaluated in this article, or claim that may be made by its manufacturer, is not guaranteed or endorsed by the publisher.

## Footnotes

1
https://phytozome.jgi.doe.gov/pz/portal.html#

2
http://pfam.xfam.org/

3
https://www.ncbi.nlm.nih.gov/Structure/bwrpsb/bwrpsb.cgi

4
http://smart.embl-heidelberg.de/

5
https://meme-suite.org/meme/tools/meme

6
https://services.healthtech.dtu.dk/service.php?TMHMM-2.0

7
https://services.healthtech.dtu.dk/service.php?SignalP-4.1

8
https://services.healthtech.dtu.dk/service.php?ChloroP-1.1

9
https://itol.embl.de/

10
http://bioinformatics.psb.ugent.be/webtools/plantcare/html/

## References

[B1] Abdullah M. Cheng X. Cao Y. Su X. Manzoor M. A. Gao J. (2018). Zinc finger-homeodomain transcriptional factors (ZHDs) in upland cotton (*Gossypium hirsutum*): genome-wide identification and expression analysis in fiber development. *Front. Genet.* 9:357. 10.3389/fgene.2018.00357 30356782 PMC6189526

[B2] Aharoni A. Luecker J. Verhoeven H. A. Van T. O’Connell A. P. (2000). *Fruit Flavour Related Genes and Use Thereof.* [Accessed on Nov 30, 2021]

[B3] Al-Dhubiab B. (2012). Pharmaceutical applications and phytochemical profile of *Cinnamomum burmannii*. *Phcog. Rev*. 6:125. 10.4103/0973-7847.99946 23055638 PMC3459454

[B4] Ao H. Wang J. Chen L. Li S. Dai C. (2019). Comparison of volatile oil between the fruits of *Amomum villosum* Lour and *Amomum villosum* Lour var xanthioides T. L. Wu et Senjen based on GC-MS and chemometric techniques. *Molecules* 24:1663. 10.3390/molecules24091663 31035329 PMC6539846

[B5] Arceusz A. Occhipinti A. Capuzzo A. Maffei M. E. (2013). Comparison of different extraction methods for the determination of α- and β-thujone in sage (*Salvia officinalis* L.) herbal tea. *J. Sep. Sci.* 36 3130–3134. 10.1002/jssc.201300206 23843295

[B6] Asghari G. Jalali M. Sadoughi E. (2012). Antimicrobial activity and chemical composition of essential oil from the seeds of *Artemisia aucheri* Boiss. *Jundishapur J. Nat. Pharm. Prod.* 7 11–15. 10.5812/kowsar.17357780.3530 24624145 PMC3941861

[B7] Bailey T. L. Elkan C. (1994). Fitting a mixture model by expectation maximization to discover motifs in biopolymers. *Proc. Int. Conf. Intell. Syst. Mol. Biol.* 2 28–36. 7584402

[B8] Balbontín C. Gaete-Eastman C. Fuentes L. Figueroa C. R. Herrera R. Manriquez D. (2010). VpAAT1, a gene encoding an alcohol acyltransferase, is involved in ester biosynthesis during ripening of mountain papaya fruit. *J. Agric. Food Chem.* 58 5114–5121. 10.1021/jf904296c 20369803

[B9] Bäumlein H. Nagy I. Villarroel R. Inzé D. Wobus U. (1992). Cis-analysis of a seed protein gene promoter: the conservative RY repeat CATGCATG within the legumin box is essential for tissue-specific expression of a legumin gene. *Plant J*. 2 233–239. 10.1046/j.1365-313x.1992.t01-45-00999.x 1338774

[B10] Beekwilder J. Alvarez-Huerta M. Neef E. Verstappen F. W. A. Bouwmeester H. J. Aharoni A. (2004). Functional characterization of enzymes forming volatile esters from strawberry and banana. *Plant Physiol*. 135 1865–1878. 10.1104/pp.104.042580 15326278 PMC520758

[B11] Chánique A. M. Dimos N. Drienovská I. Calderini E. Pantín M. P. Helmer C. P. O. (2021). A structural view on the stereospecificity of plant borneol-type dehydrogenases. *ChemCatChem* 13 2262–2277. 10.1002/cctc.202100110 34262629 PMC8261865

[B12] Chen L. X. Lai Y.-F. Zhang W.-X. Cai J. Hu H. Wang Y. (2020b). Comparison of volatile compounds in different parts of fresh *Amomum villosum Lour.* from different geographical areas using cryogenic grinding combined HS-SPME-GC-MS. *Chin. Med*. 15:97. 10.1186/s13020-020-00377-z 32944063 PMC7487758

[B13] Chen C. Chen H. Zhang Y. Thomas H. R. Frank M. H. He Y. (2020a). TBtools: an integrative toolkit developed for interactive analyses of big biological data. *Mol. Plant* 13 1194–1202. 10.1016/j.molp.2020.06.009 32585190

[B14] Commission of Chinese Materia Medica. (1999). *Chinese Materia Medica, Vol. 8.* Shanghai: Shanghai Science and Technology Publishing House.

[B15] Commission of Chinese Pharmacopoeia. (2015). *Pharmacopoeia of the People’s Republic of China.* Beijing: China Medico-Pharmaceutical Science.

[B16] Croteau R. Hooper C. L. (1978). Metabolism of monoterpenes: acetylation of (-)-menthol by a soluble enzyme preparation from peppermint (*Mentha piperita*) leaves. *Plant Physiol*. 61 737–742. 10.1104/pp.61.5.737 16660375 PMC1091967

[B17] Cumplido-Laso G. Medina-Puche L. Moyano E. Hoffmann T. Sinz Q. Ring L. (2012). The fruit ripening-related gene FaAAT2 encodes an acyl transferase involved in strawberry aroma biogenesis. *J. Exp. Bot.* 63 4275–4290. 10.1093/jxb/ers120 22563120

[B18] Cutillas A.-B. Carrasco A. Martinez-Gutierrez R. Tomas V. Tudela J. (2018). Thymus mastichina L. essential oils from Murcia (Spain): composition and antioxidant, antienzymatic and antimicrobial bioactivities. *PLos One* 13:e0190790. 10.1371/journal.pone.0190790 29304179 PMC5755899

[B19] D’Auria J. C. (2006). Acyltransferases in plants: a good time to be BAHD. *Curr. Opin. Plant Biol.* 9 331–340. 10.1016/j.pbi.2006.03.016 16616872

[B20] Despinasse Y. Fiorucci S. Antonczak S. Moja S. Bony A. Nicolè F. (2017). Bornyl-diphosphate synthase from *Lavandula angustifolia*: a major monoterpene synthase involved in essential oil quality. *Phytochemistry* 137 24–33. 10.1016/j.phytochem.2017.01.015 28190677

[B21] Dhar N. Sarangapani S. Reddy V. A. Kumar N. Panicker D. Jin J. (2020). Characterization of a sweet basil acyltransferase involved in eugenol biosynthesis. *J. Exp. Bot.* 71 3638–3652. 10.1093/jxb/eraa142 32198522 PMC7307857

[B22] Drienovská I. Kolanoviæ D. Chánique A. Sieber V. Hofer M. Kourist R. (2020). Molecular cloning and functional characterization of a two highly stereoselective borneol dehydrogenases from *Salvia officinalis* L. *Phytochemistry* 172:112227. 10.1016/j.phytochem.2019.112227 31927319

[B23] Ellerström M. Stålberg K. Ezcurra I. Rask L. (1996). Functional dissection of a napin gene promoter: identification of promoter elements required for embryo and endosperm-specific transcription. *Plant Mol. Biol.* 32 1019–1027. 10.1007/BF00041385 9002600

[B24] Emanuelsson O. Nielsen H. von Heijne G. (1999). ChloroP, a neural network-based method for predicting chloroplast transit peptides and their cleavage sites. *Protein Sci.* 8 978–984. 10.1110/ps.8.5.978 10338008 PMC2144330

[B25] Fidan H. Stefanova G. Kostova I. Stankov S. Damyanova S. Stoyanova A. (2019). Chemical composition and antimicrobial activity of *Laurus nobilis* L essential oils from Bulgaria. *Molecules* 24:804. 10.3390/molecules24040804 30813368 PMC6412751

[B26] Hoffmann L. Maury S. Martz F. Geoffroy P. Legrand M. (2003). Purification, cloning, and properties of an acyltransferase controlling shikimate and quinate ester intermediates in phenylpropanoid metabolism. *J. Biol. Chem.* 278 95–103. 10.1074/jbc.M209362200 12381722

[B27] Hurd M. C. Kwon M. Ro D.-K. (2017). Functional identification of a *Lippia dulcis* bornyl diphosphate synthase that contains a duplicated, inhibitory arginine-rich motif. *Biochem. Biophys. Res. Commun.* 490 963–968. 10.1016/j.bbrc.2017.06.147 28655616

[B28] Izawa T. Foster R. Chua N. H. (1993). Plant bZIP protein DNA binding specificity. *J. Mol. Biol.* 230 1131–1144. 10.1006/jmbi.1993.1230 8487298

[B29] Izawa T. Foster R. Nakajima M. Shimamoto K. Chua N. H. (1994). The rice bZIP transcriptional activator RITA-1 is highly expressed during seed development. *Plant Cell* 6 1277–1287. 10.1105/tpc.6.9.1277 7919992 PMC160519

[B30] Khine A. A. Yang M.-Y. Hu A. Lin G.-H. Toh Y.-H. Chen H.-P. (2020). Production of optically pure (–)-borneol by *Pseudomonas monteilii* TCU-CK1 and characterization of borneol dehydrogenase involved. *Enzyme Microb. Technol.* 139:109586. 10.1016/j.enzmictec.2020.109586 32732035

[B31] Krogh A. Larsson B. von Heijne G. Sonnhammer E. L. L. (2001). Predicting transmembrane protein topology with a hidden markov model: application to complete genomes11 Edited by F. *Cohen. J. Mol. Biol.* 305 567–580. 10.1006/jmbi.2000.4315 11152613

[B32] Kumar G. Kumar P. Kapoor R. Lore J. S. Bhatia D. Kumar A. (2021). Characterization of evolutionarily distinct rice BAHD-Acyltransferases provides insight into their plausible role in rice susceptibility to *Rhizoctonia solani*. *Plant Genome* 14:e20140. 10.1002/tpg2.20140 34498798 PMC12807277

[B33] Kumar S. Stecher G. Li M. Knyaz C. Tamura K. (2018). MEGA X: molecular evolutionary genetics analysis across computing platforms. *Mol. Biol. Evol.* 35 1547–1549. 10.1093/molbev/msy096 29722887 PMC5967553

[B34] Lescot M. Déhais P. Thijs G. Marchal K. Moreau Y. Van de Peer Y. (2002). PlantCARE, a database of plant cis-acting regulatory elements and a portal to tools for in silico analysis of promoter sequences. *Nucleic Acids Res.* 30 325–327. 10.1093/nar/30.1.325 11752327 PMC99092

[B35] Letunic I. Bork P. (2021). Interactive tree of life (iTOL) v5: an online tool for phylogenetic tree display and annotation. *Nucleic Acids Res.* 49 W293–W296. 10.1093/nar/gkab301 33885785 PMC8265157

[B36] Lin J.-L. Zhu J. Wheeldon I. (2016). Rapid ester biosynthesis screening reveals a high activity alcohol-O-acyltransferase (AATase) from tomato fruit. *Biotechnol. J.* 11 700–707. 10.1002/biot.201500406 26814045

[B37] Liu C. Qiao X. Li Q. Zeng W. Wei S. Wang X. (2020). Genome-wide comparative analysis of the BAHD superfamily in seven *Rosaceae* species and expression analysis in pear (*Pyrus bretschneideri*). *BMC Plant Biol.* 20:14. 10.1186/s12870-019-2230-z 31914928 PMC6950883

[B38] Liu P. Liu X.-C. Dong H.-W. Liu Z.-L. Du S.-S. Deng Z.-W. (2012). Chemical composition and insecticidal activity of the essential oil of *Illicium pachyphyllum* fruits against two grain storage insects. *Molecules* 17 14870–14881. 10.3390/molecules171214870 23519259 PMC6268823

[B39] Ma R. Su P. Guo J. Jin B. Ma Q. Zhang H. (2021). Bornyl diphosphate synthase from *Cinnamomum burmanni* and its application for (+)-borneol biosynthesis in Yeast. *Front. Bioeng. Biotechnol*. 9:631863. 10.3389/fbioe.2021.631863 33644023 PMC7905068

[B40] Ma X. Koepke J. Panjikar S. Fritzsch G. Stöckigt J. (2005). Crystal structure of vinorine synthase, the first representative of the BAHD superfamily. *J. Biol. Chem.* 280 13576–13583. 10.1074/jbc.M414508200 15665331

[B41] Minetoki T. Bogaki T. Iwamatsu A. Fujii T. Hamachi M. (1993). The purification, properties and internal peptide sequences of alcohol acetyltransferase isolated from *Saccharomyces cerevisiae* Kyokai No. 7. *Biosci. Biotechnol. Biochem.* 57 2094–2098. 10.1271/bbb.57.2094 7764365

[B42] Nimitkeatkai H. Shishido M. Okawa K. Ohara H. Ban Y. Kita M. (2011). Effect of jasmonates on ethylene biosynthesis and aroma volatile emission in Japanese apricot infected by a pathogen (*Colletotrichum gloeosporioides*). *J. Agric. Food Chem.* 59 6423–6429. 10.1021/jf2010996 21599017

[B43] Ohtsubo S. Fujita T. Matsushita A. Kumamoto E. (2015). Inhibition of the compound action potentials of frog sciatic nerves by aroma oil compounds having various chemical structures. *Pharmacol. Res. Perspect.* 3:e00127. 10.1002/prp2.127 26038703 PMC4448976

[B44] Onodera Y. Suzuki A. Wu C. Y. Washida H. Takaiwa F. (2001). A rice functional transcriptional activator, RISBZ1, responsible for endosperm-specific expression of storage protein genes through GCN4 motif. *J. Biol. Chem.* 276 14139–14152. 10.1074/jbc.M007405200 11133985

[B45] Perestrelo R. Silva C. L. Rodrigues F. Caldeira M. Câmara J. S. (2016). A powerful approach to explore the potential of medicinal plants as a natural source of odor and antioxidant compounds. *J. Food Sci. Technol.* 53 132–144. 10.1007/s13197-015-2022-x 26787937 PMC4711449

[B46] Petersen T. N. Brunak S. von Heijne G. Nielsen H. (2011). SignalP 4.0: discriminating signal peptides from transmembrane regions. *Nat. Methods* 8 785–786. 10.1038/nmeth.1701 21959131

[B47] Petrik D. L. Karlen S. D. Cass C. L. Padmakshan D. Lu F. Liu S. (2014). p-Coumaroyl-CoA: monolignol transferase (PMT) acts specifically in the lignin biosynthetic pathway in *Brachypodium distachyon*. *Plant J.* 77 713–726. 10.1111/tpj.12420 24372757 PMC4282527

[B48] Potter S. C. Luciani A. Eddy S. R. Park Y. Lopez R. Finn R. D. (2018). HMMER web server: 2018 update. *Nucleic Acids Res.* 46 W200–W204. 10.1093/nar/gky448 29905871 PMC6030962

[B49] Raina A. P. Negi K. S. (2015). Essential oil composition of *Valeriana Jatamansi* Jones from himalayan regions of India. *Indian J. Pharm. Sci.* 77 218–222. 10.4103/0250-474x.156614 26009656 PMC4442472

[B50] Reyes-Sánchez F. J. Páez-Lerma J. B. Rojas-Contreras J. A. López-Miranda J. Soto-Cruz Ó Reinhart-Kirchmayr M. (2019). Study of the enzymatic capacity of *Kluyveromyces marxianus* for the synthesis of esters. *J. Mol. Microbiol. Biotechnol.* 29 1–9. 10.1159/000507551 32325454

[B51] Sajjadi S.-E. Jamali M. Shokoohinia Y. Abdi G. Shahbazi B. Fattahi A. (2015). Antiproliferative evaluation of terpenoids and terpenoid coumarins from *Ferulago macrocarpa* (Fenzl) Boiss. fruits. *Pharmacognosy Res.* 7 322–328. 10.4103/0974-8490.158437 26692745 PMC4660510

[B52] Sarker L. S. Mahmoud S. S. (2015). Cloning and functional characterization of two monoterpene acetyltransferases from glandular trichomes of *L. x intermedia*. *Planta* 242 709–719. 10.1007/s00425-015-2325-1 25998527

[B53] Shalit M. Guterman I. Volpin H. Bar E. Tamari T. Menda N. (2003). Volatile ester formation in roses. Identification of an acetyl-coenzyme A. Geraniol/Citronellol acetyltransferase in developing rose petals. *Plant Physiol.* 131 1868–1876. 10.1104/pp.102.018572 12692346 PMC166943

[B54] Souleyre E. J. F. Chagné D. Chen X. Tomes S. Turner R. M. Wang M. Y. (2014). The AAT1 locus is critical for the biosynthesis of esters contributing to ‘ripe apple’ flavour in ‘Royal Gala’ and ‘Granny Smith’ apples. *Plant J.* 78 903–915. 10.1111/tpj.12518 24661745

[B55] Souleyre E. J. F. Greenwood D. R. Friel E. N. Karunairetnam S. Newcomb R. D. (2005). An alcohol acyl transferase from apple (cv. Royal Gala), MpAAT1, produces esters involved in apple fruit flavor: characterization of apple alcohol acyl transferase. *FEBS J.* 272 3132–3144. 10.1111/j.1742-4658.2005.04732.x 15955071

[B56] St-Pierre B. De Luca V. (2000). “Chapter Nine Evolution of acyltransferase genes: origin and diversification of the BAHD superfamily of acyltransferases involved in secondary metabolism,” in *Recent Advance Phytochem Evolution of Metabolic Pathways*, eds Romeo J. T. Ibrahim R. Varin L. De Luca V. (Netherlands: Elsevier), 285–315. 10.1016/S0079-9920(00)80010-6

[B57] Vranová E. Coman D. Gruissem W. (2013). Network analysis of the MVA and MEP pathways for isoprenoid synthesis. *Annu. Rev. Plant Biol.* 64 665–700. 10.1146/annurev-arplant-050312-120116 23451776

[B58] Wang H. Ma D. Yang J. Deng K. Li M. Ji X. (2018). An integrative volatile terpenoid profiling and transcriptomics analysis for gene mining and functional characterization of AvBPPS and AvPS involved in the monoterpenoid biosynthesis in *Amomum villosum*. *Front. Plant Sci.* 9:846. 10.3389/fpls.2018.00846 29973947 PMC6020762

[B59] Wang X. Ma A. Shi W. Geng M. Zhong X. Zhao Y. (2011). Quercetin and bornyl acetate regulate T-lymphocyte subsets and INF- γ /IL-4 ratio I in pregnant mice. *Evid. Based Complement. Alternat. Med.* 2011:745262. 10.1155/2011/745262 20981318 PMC2958556

[B60] Wang Y. Tang H. Debarry J. D. Tan X. Li J. Wang X. (2012). MCScanX: a toolkit for detection and evolutionary analysis of gene synteny and collinearity. *Nucleic Acids Res.* 40:e49. 10.1093/nar/gkr1293 22217600 PMC3326336

[B61] Waterhouse A. M. Procter J. B. Martin D. M. A. Clamp M. Barton G. J. (2009). Jalview Version 2–a multiple sequence alignment editor and analysis workbench. *Bioinformatics* 25 1189–1191. 10.1093/bioinformatics/btp033 19151095 PMC2672624

[B62] Wise M. L. Savage T. J. Katahira E. Croteau R. (1998). Monoterpene synthases from common sage (*Salvia officinalis*). *J. Biol. Chem.* 273 14891–14899. 10.1074/jbc.273.24.14891 9614092

[B63] Yan X. Qin X. Li W. Liang D. Qiao J. Li Y. (2020). Functional characterization and catalytic activity improvement of BAHD acyltransferase from *Celastrus angulatus* Maxim. *Planta* 252:6. 10.1007/s00425-020-03413-2 32556997

[B64] Zhang B. Shen J. Wei W. Xi W. Xu C.-J. Ferguson I. (2010). Expression of genes associated with aroma formation derived from the fatty acid pathway during peach fruit ripening. *J. Agric. Food Chem.* 58 6157–6165. 10.1021/jf100172e 20415420

[B65] Zhang T. Lu S. H. Bi Q. Liang L. Wang Y. F. Yang X. X. (2017). Volatile oil from Amomi Fructus attenuates 5-fluorouracil-induced intestinal mucositis. *Front. Pharmacol.* 8:786. 10.3389/fphar.2017.00786 29170638 PMC5684147

[B66] Zhao H. Li M. Zhao Y. Lin X. Liang H. Wei J. (2021b). A comparison of two monoterpenoid synthases reveals molecular mechanisms associated with the difference of bioactive monoterpenoids between *Amomum villosum* and *Amomum longiligulare*. *Front. Plant Sci.* 12:695551. 10.3389/fpls.2021.695551 34475877 PMC8406774

[B67] Zhao H. Li M. Lin X. Zhao Yuan Y. Liang H. Ye Z. (2021a). Cloning and bioinformatics analysis of promoters of three terpene synthase genes and their terpenoid regulation in *Amomum villosum*. *Chinese Tradit. Herb Drugs* 52 1117–1127.

[B68] Zhu J. Lin J.-L. Palomec L. Wheeldon I. (2015). Microbial host selection affects intracellular localization and activity of alcohol-O-acetyltransferase. *Microb. Cell Fact.* 14:35. 10.1186/s12934-015-0221-9 25880435 PMC4367896

