# Supplementary material for: Genome-Wide Identification of BAHD Superfamily and Functional Characterization of Bornyl Acetyltransferases Involved in the Bornyl Acetate Biosynthesis in Wurfbainia villosa
Source: Front Plant Sci. 2022 Mar 28;13:860152. doi: 10.3389/fpls.2022.860152 (PMC9011770; doi:10.3389/fpls.2022.860152)
Supplement: Supplementary file 1 [file Data_Sheet_1.docx]

**
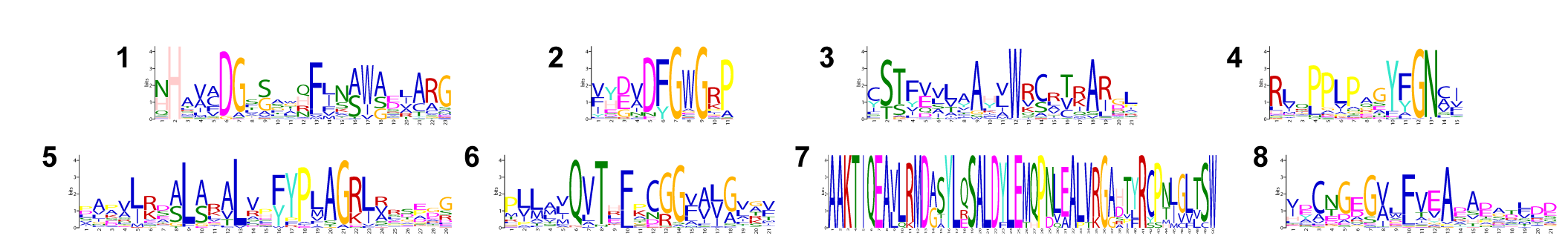
**

**Supplementary Figure 1 |** Legend depicting the amino acid sequence of the corresponding motif.


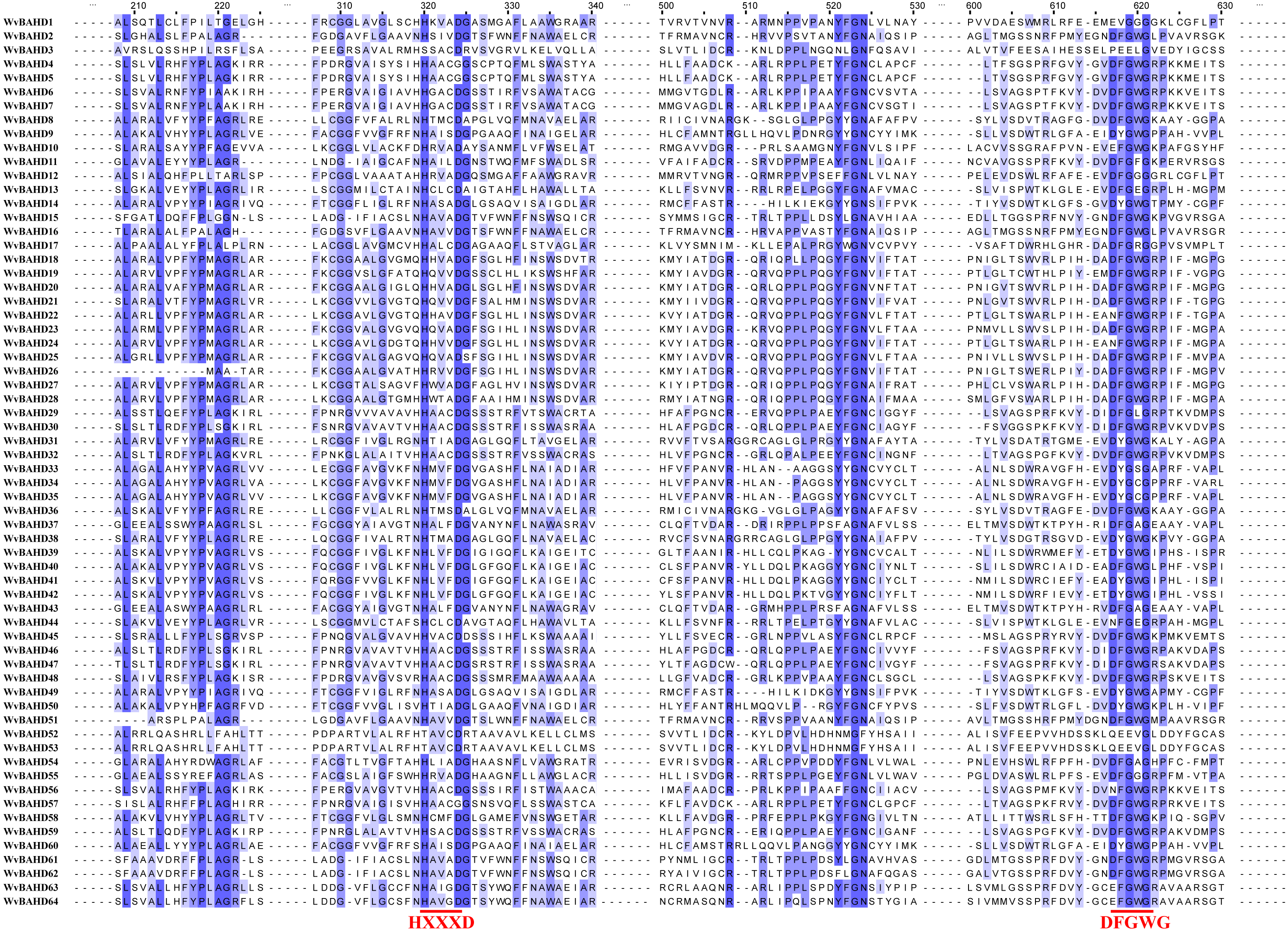


**Supplementary Figure 2 |** Sequence alignment of the members of BAHD gene family in *W. villosa*. Percentage identify threshold was 30% or above. WvBAHD3, 52 and 53 were screened out using PF07247 (AATase), and the rest were screened with PF02458. Conserved motifs were shown above the red lines.


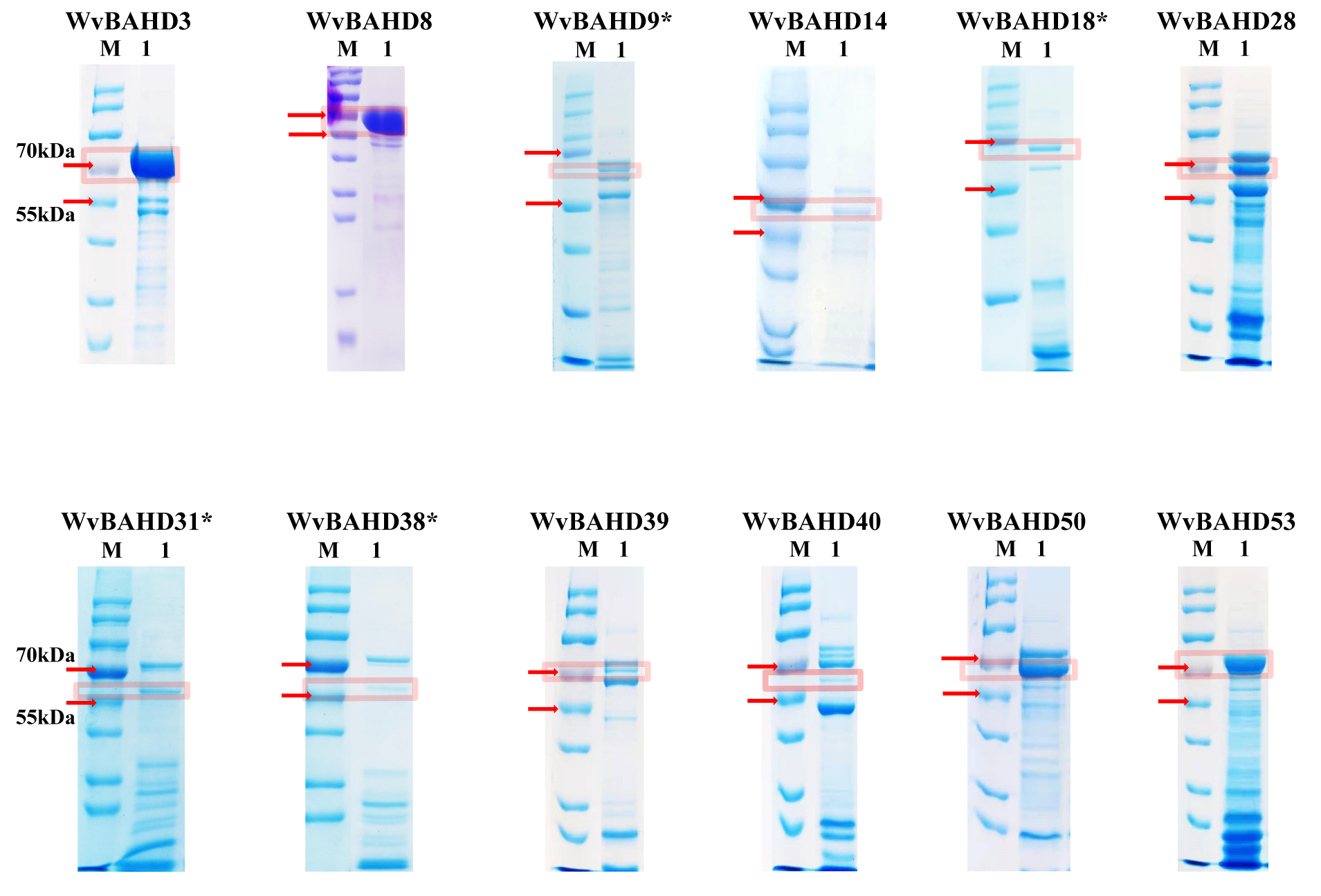


**Supplementary Figure 3 |** SDS-PAGE analysis of (A) each WvAAT candidate recombinant protein expressed in Escherichia coli Rosetta (DE3) harboring pET32a-WvAAT. Lanes M and 1 indicated marker and purified WvAAT recombinant protein, respectively. Red box indicates target protein. Protein without catalytic activity were indicated by an asterisk.


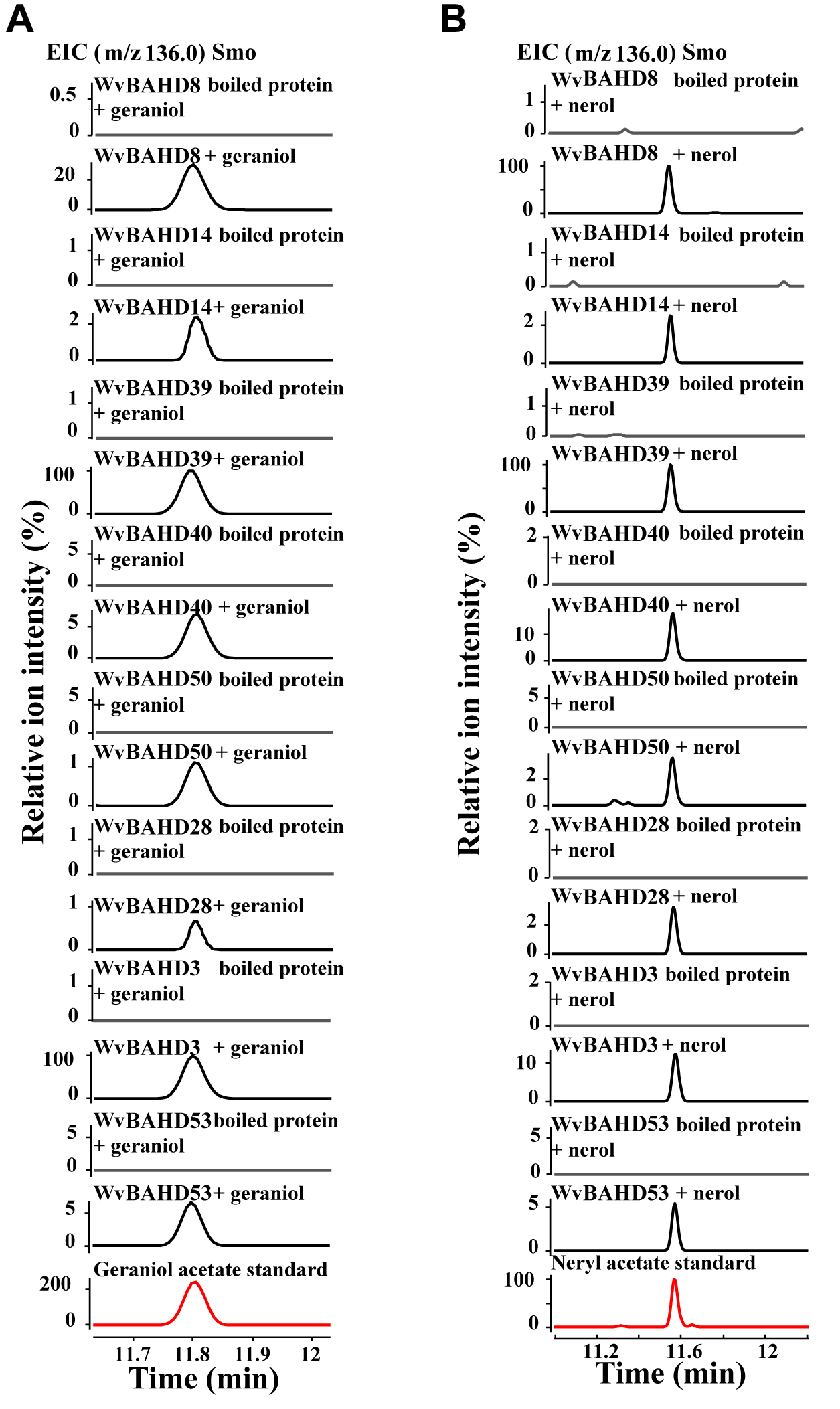


**Supplementary Figure 4 |** Functional characterization of candidate WvAATs. (A and B) the GC-MS chromatogram of the *in vitro* reaction products yielded by WvBAHDs using geraniol and nerol as the substrate, respectively.


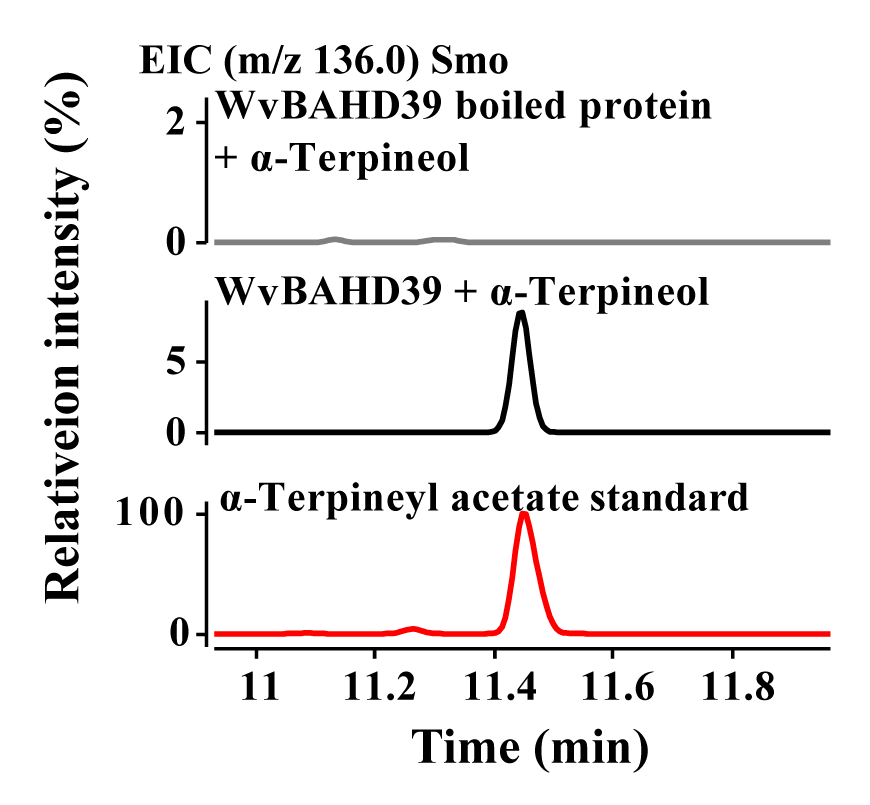


**Supplementary Figure 5 |**Functional characterization of WvBAHD39. The GC-MS chromatogram of the *in vitro* reaction products by WvBAHD39 using α-terpineol as the substrate.


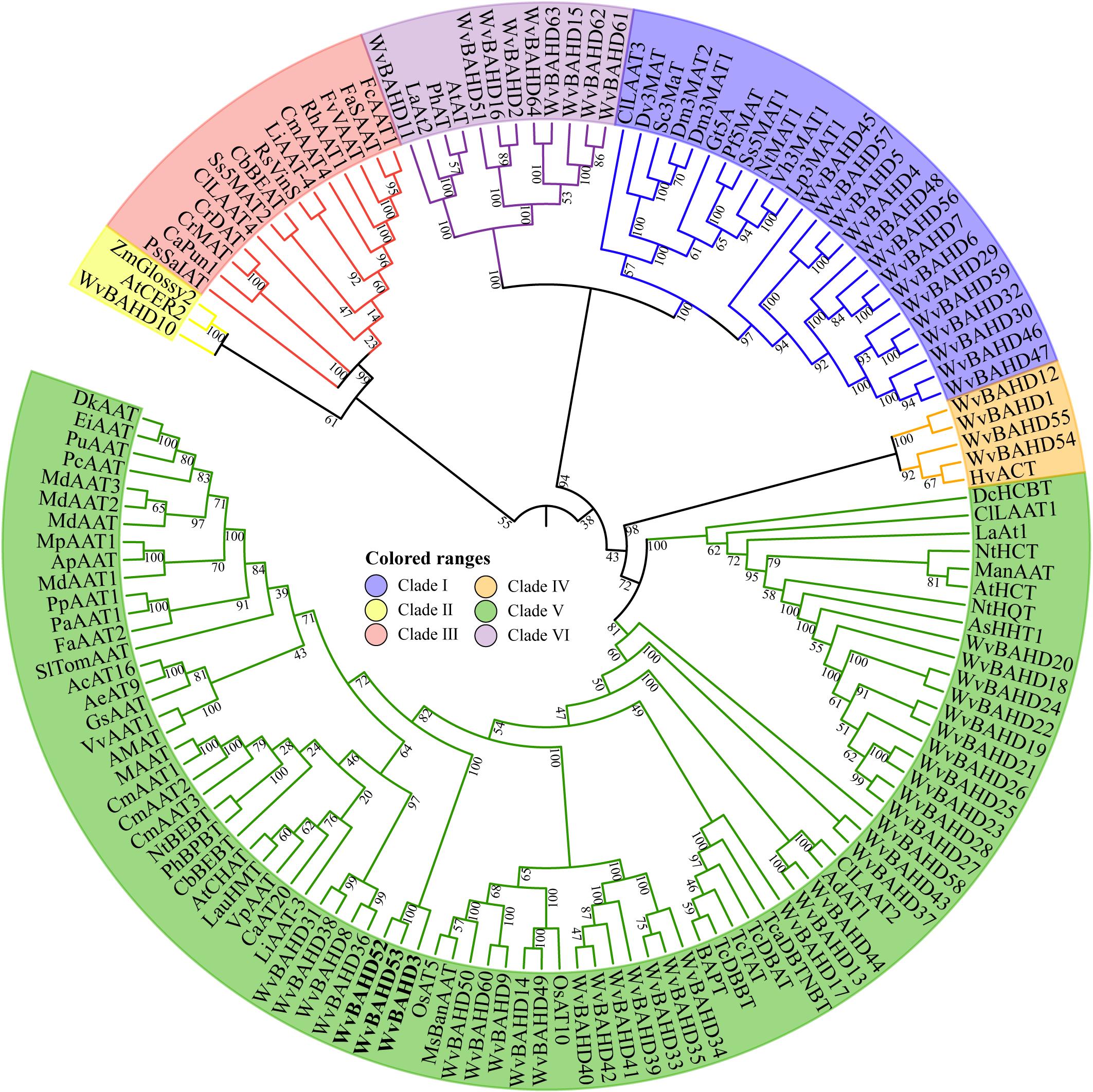


**Supplementary Figure 6 |** Phylogenetic tree analysis of identified BAHD acyltransferases including WvBAHD candidates. The tree was constructed by maximum-likelihood and bootstrap (1000 replicates) distance analysis, and the clade of clustering adapted from D’Auria 2006; Sarker and Mahmoud, 2015). Three AATase-WvBATs were indicated in bold. The GenBank accession numbers of each BAHD acyltransferase were listed in **supplementary table S8**.
