# Supplementary material for: Genome-Wide Identification of BAHD Superfamily and Functional Characterization of Bornyl Acetyltransferases Involved in the Bornyl Acetate Biosynthesis in Wurfbainia villosa
Source: Front Plant Sci. 2022 Mar 28;13:860152. doi: 10.3389/fpls.2022.860152 (PMC9011770; doi:10.3389/fpls.2022.860152)
Supplement: Supplementary file 2 [file Data_Sheet_2.docx]

**Supplementary Table 1. Terpene alcohols and acetates in *Wurfbainia villosa***

| **Terpene alcohol** | **Terpene acetate** | **Reference** |
| --- | --- | --- |
| Borneol | Bornyl acetate | Wang et al., 2018; Zhao et al., 2021b |
| Isoborneol | Isobornyl acetate | Zhao et al., 2021b; Huang, 2020 |
| Myrtenol | Myrtenyl acetate | Huang, 2020; Chen et al., 2020b |
| Santalol | Santalyl acetate | Chen et al., 2020b; Zhao et al., 2021b |
| Nerolidol | Nerolidyl acetate | Chen et al., 2020b |
| - | Fenchyl acetate | Chen et al., 2020b; Zhao et al., 2021b |
| - | Isopulegol acetate | Huang, 2020 |
| α-Terpineol | - | Wang et al., 2018 |
| Terpinen-4-ol | - | Zhao et al., 2021b |
| Cubenol | - | Chen et al., 2020b |
| Linalool | - | Huang, 2020 |
| Espatulenol | - | Wang et al., 2018 |

**Supplementary Table 2.** **The information of the reported alcohol acyltransferases from other species**

| **Acyltransferase name** | | **NCBI GenBank**  **ID number** | **Alcohol substrate** | **Major Acyl-CoA donor** | **Major product** | **Tissue** | **Reference** |
| --- | --- | --- | --- | --- | --- | --- | --- |
| MsBanAAT | Banana (*Musa sapientum*) alcohol acyltransferase | CAC09063 | Cinnamyl alcohol ,geraniol,  nerol, and the C6 and C8 alcohols | Acetyl-CoA, hexanoyl-CoA, butanoyl-CoA | Cinnamyl acetate and other medium-chain aliphatic acetate and esters (isoamyl acetate, hexyl acetate, isoamyl butyrate , etc.) | Pulp | Beekwilder et al., 2004 |
| LiAAT-3 | *Lavandula. x intermedia*  alcohol acetyltransferase | AIW81431 | Terpene alcohols  (geraniol, lavandulol, and nerol) | Acetyl-CoA | Geranyl acetate, lavandulyl acetate, and neryl acetate | Glandular trichome, | Sarker and Mahmoud, 2015 |
| FaAAT2 | *Fragaria x ananassa* alcohol acyltransferase | AEM43830 | C1-C8 linear alcohols, aromatic alcohols (cinnamyl alcohol, geraniol, and eugenol , etc.) | Acetyl-CoA, propionyl-CoA | Cinnamyl, hexyl and geranyl acetate , etc. | Fruit receptacle | Cumplido-Laso et al., 2012 |
| VpAAT1 | *Vasconellea Pubescens* alcohol acyltransferase | FJ548611 | Benzyl alcohol, Cinnamyl alcohol, geraniol, methanol, butanol , etc. | Acetyl-CoA,  hexanoyl-CoA,  butanoyl-CoA | Benzyl acetate, cinnamyl and geranyl acetate; methy hexanoate; butyl butanoate , etc. | Whole fruit | Balbontín et al., 2010 |
| ClLAAT  1-4 | Lemon (*Citrus limon*) acyltransferase | CAC09049  CAC09067  CAC09066  CAC09065 | Butanol, geraniol, nerol, citronellol | Acetyl-CoA | Butyl acetate, geranyl acetate,  neryl acetate, citronellyl acetate | Fruit peel | Aharoni et al., 2000 |
| CaAT20 | *Celastrus angulatus* BAHD acyltransferase | QLI57449 | Geraniol | Benzoyl-CoA,  acetyl-CoA | Geranyl benzoate,  geranyl acetate | -- | Yan et al., 2020 |
| RhAAT1 | *Rosa hybrida* acetyl CoA geraniol /citronellol acetyltransferase | AAW31948 | Geraniol, citronellol and  2-phenylethyl alcohol and cis-3-hexene 1-alcohol , etc. | Acetyl-CoA | Geranyl acetate , citronellyl acetate and other volatile esters | Petal | Shalit et al., 2003 |
| FaSAAT | Strawberry (*Fragaria*  *x ananassa*)alcohol acyltransferase | AAG13130 | C6 to C9 aliphatic alcohols, terpene alcohols , etc. | Acetyl-CoA, hexanoyl-CoA,  butanoyl-CoA | Medium-chain aliphatic, benzyl esters (methyl hexanoate, hexyl butyrate) and geranyl acetate , etc. | Whole fruit | Aharoni et al., 2000 |
| FvVAAT | *Fragaria vesca* alcohol acyltransferase | CAC09062 | C6 aliphatic alcohols,  terpene alcohols , etc. | Acetyl-CoA, hexanoyl-CoA,  butanoyl-CoA | Small- to medium-chain aliphatic esters (ethyl acetate, ethyl butanoate, ethyl hexanoate, octyl acetate), geranyl acetate , etc. | Whole fruit | Beekwilder et al., 2004 |
| LiAAT-4 | *Lavandula. x intermedia*  alcohol acetyltransferase | AIW81432 | Terpene alcohols(geraniol, lavandulol, and nerol) | Acetyl-CoA | Geranyl acetate, lavandulyl acetate,and neryl acetate | Glandular trichome amd flower | Sarker and Mahmoud, 2015 |
| CmAAT4 | *Cucumis melo* alcohol acyltransferase | AAW51126 | Medium-chain aliphatic(Cinnamyl alcohol), geraniol | Acetyl-CoA, Butanoyl-CoA | Medium-chain and  Hydroxycinnamoyl acyl esters, geranyl acetate | Whole fruit | El-Sharkawy et al., 2005 |

| **Supplementary Table 3. Primers used in this study** | | |  |
| --- | --- | --- | --- |
| **Name** | **Primer (5’--> 3’)** | **Function** | |
| WvBAHD8 | 1^st^ F: CACGGCGGGGAAGCTTTC | For gene cloning | |
|  | 1^st^ R: CTCCTCCCCCTTGCTGTTCTTC |  |  |
|  | 2^nd^ F: GCAGCTGCTTGTTTCTACTG |  |  |
|  | 2^nd^ R: GCTTAGGATCGGAGCTCAAGT |  |  |
| WvBAHD14 | 1^st^ F: GCAGGTAGGAGATGAGCATGG |  |  |
|  | 1^st^ R: CTATTCAAGTGGAAAATCAATTTGTCCA |  |  |
|  | 2^nd^ F: ATGAGCATGGCGTCTTGG |  |  |
|  | 2^nd^ R: CTATTCAAGTGGAAAATCAATTTGTCC |  |  |
| WvBAHD39 | F: GAACACACGATTTTTCTTTATTCACTTGT |  |  |
|  | R: CTATTCAAGTGGAAAATCAATTTGTCCA |  |  |
| WvBAHD40 | F: ATGAGCATGGCGTCCT |  |  |
|  | R: TCACAACATAACATTAGCTAAGAATTTATT |  |  |
| WvBAHD28 | F: AGCGGTCATTAATTTGCGGTG |  |  |
|  | R: GCCTGCTTTCAGATTGTCGT |  |  |
| WvBAHD50 | 1^st^ F: CGCAACTTTTCTCTGCTCCAC |  |  |
|  | 1^st^ R: TTATGCAGCATGCACCCCGT |  |  |
|  | 2^nd^ F: AGGCCACCAACCATGAGCTT |  |  |
|  | 2^nd^ R: TTATGCAGCATGCACCCCGT |  |  |
| WvBAHD3 | F: GTACAAACCTTTGCTTGTTCGC |  |  |
|  | R: CTACTCTTCTTCGCTTCCCAAAA |  |  |
| WvBAHD53 | F: ATGTCTGAGGCGGATGCC |  |  |
|  | R: TCAATATTTCTCAATCTCTTTCGATTCAC |  |  |
| pET32a-WvBAHD8 | F:TGGCTGATATCGGATCCGAATTCATGGCATCCTCCTCCCTCAC | For construction of expression vectors | |
|  | R:AGTGCGGCCGCAAGCTTGTCGACAAGTGCAGAGACAATGAGGACG |  |  |
| pET32a-WvBAHD14 | F:CCATGGCTGATATCGGATCCGAATTCATGGGCTTCACCGTCACG |  |  |
|  | R:TGGTGCTCGAGTGCGGCCGCAAGCTTGTCGAACAGGTTAACTCGCTCG |  |  |
| pET32a-WvBAHD39 | F:TGATATCGGATCCGAATTCATGCAGGAGATGATTTCCGTGT |  |  |
|  | R:CCGCAAGCTTGTCGACGGAGCTCTTCAAGTGGAAAATCAATTTGTCCATTATGAGATACC |  |  |
| pET32a-WvBAHD40 | F:TATCGGATCCGAATTCATGAGCATGGCGTCCTGG |  |  |
|  | R:GTGCGGCCGCAAGCTTCAACATAACATTAGCTAAGAATTTATTAATCTCATCAATAAATCTTTT |  |  |
| pET32a-WvBAHD28 | F:CCATGGCTGATATCGGATCCGAATTCATGGTGGTGATCAACGTGCG |  |  |
|  | R:TGGTGCTCGAGTGCGGCCGCAAGCTTGATATCATAGATGAGCTCCTGGAACTTCAC |  |  |
| pET32a- WvBAHD50 | F:TGGCTGATATCGGATCCGAATTCATGAGCTTCTCCGTCACCAG |  |  |
|  | R:GCGGCCGCAAGCTTGTCGACGGAGCTCTGCAGCATGCACCCCG |  |  |
| pET32a- WvBAHD3 | F:TGGCTGATATCGGATCCGAATTCATGGCCTCCGGCGAAG |  |  |
|  | R:GCGGCCGCAAGCTTGTCGACGGAGCTCCTCTTCTTCGCTTCCCAAAAGGA |  |  |
| pET32a- WvBAHD53 | F:TGGCTGATATCGGATCCGAATTCATGTCTGAGGCGGATGCC |  |  |
|  | R:GCGGCCGCAAGCTTGTCGACGGAGCTCATATTTCTCAATCTCTTTCGATTCACCATTTAATGTG |  |  |
| qRT-WvBAHD8 | F: CACGGCGGGGAAGCTTTC | For qRT-PCR | |
|  | R: CTCCTCCCCCTTGCTGTTCTTC |  |  |
| qRT-WvBAHD14 | F: TTGAAGTGGGCTAATGACGAATTTGAG |  |  |
|  | R: TGGTGCCGGTGCCTTCAAGAGA |  |  |
| qRT-WvBAHD39 | F: GAACACACGATTTTTCTTTATTCACTTGT |  |  |
|  | R: CTATTCAAGTGGAAAATCAATTTGTCCA |  |  |
| qRT-WvBAHD40 | F: ATCCGGCAGCGAAGGTCAT |  |  |
|  | R: AAGGACAACCGGGAATCAGCTCG |  |  |
| qRT-WvBAHD28 | F: ACCGGCATGCATCATTGGAC |  |  |
|  | R: GAGGACAAGGAAGGAAAGGGCA |  |  |
| qRT- WvBAHD50 | F: GAGGTCATCCCCAGTCCTCC |  |  |
|  | R: CTGCATCAGATGGCGGGTG |  |  |
| qRT- WvBAHD3 | F: TTTGCGGAGCAATCGGAGCT |  |  |
|  | R: CTCATGGCATCTGCCCACGA |  |  |
| qRT- WvBAHD53 | F: CACAATTAACCCCTTCATCATCCCTA |  |  |
|  | R: CTCTTTCGATTCACCATTTAATGTGC |  |  |
| WvTUA | F: GGAGGATGCGGCAAACAA |  |  |
|  | R: AGCAAGGAACCCAGCCCAGA |  |  |

The second round of PCR for cloning genes used the first round of PCR product as a template.

**Supplementary Table 4. WvBAHDs information**

| **No.** | **Gene name** | **Original**  **gene ID** | **Chromosome/**  **Contig location** | **Starting site** | **Termination site** | **Protein size(aa)** | **BAHD clade** |
| --- | --- | --- | --- | --- | --- | --- | --- |
| 1 | WvBAHD1 | Wv_025531 | LG02 | 138133486 | 138134918 | 441 | Ⅰ-b |
| 2 | WvBAHD2 | Wv_032466 | LG06 | 993192 | 994664 | 490 | Ⅱ-a |
| 3 | WvBAHD3 | Wv_032842 | LG06 | 4902700 | 4905873 | 488 | Ⅵ |
| 4 | WvBAHD4 | Wv_033324 | LG06 | 8663681 | 8665048 | 455 | Ⅱ-b |
| 5 | WvBAHD5 | Wv_033327 | LG06 | 8678430 | 8679842 | 470 | Ⅱ-b |
| 6 | WvBAHD6 | Wv_033328 | LG06 | 8686721 | 8688121 | 466 | Ⅱ-b |
| 7 | WvBAHD7 | Wv_033330 | LG06 | 8697152 | 8698525 | 457 | Ⅱ-b |
| 8 | WvBAHD8 | Wv_035278 | LG06 | 29108278 | 29109654 | 458 | Ⅰ-a |
| 9 | WvBAHD9 | Wv_038114 | LG06 | 52492001 | 52493728 | 424 | Ⅰ-a |
| 10 | WvBAHD10 | Wv_042053 | LG07 | 120765849 | 120767328 | 446 | Ⅲ-a |
| 11 | WvBAHD11 | Wv_042071 | LG07 | 120840928 | 120842336 | 444 | Ⅱ-a |
| 12 | WvBAHD12 | Wv_046304 | LG09 | 114260137 | 114261432 | 431 | Ⅰ-b |
| 13 | WvBAHD13 | Wv_046482 | LG09 | 115990482 | 115991971 | 443 | Ⅰ-a |
| 14 | WvBAHD14 | Wv_049056 | LG11 | 55405292 | 55407556 | 411 | Ⅰ-a |
| 15 | WvBAHD15 | Wv_050720 | LG11 | 101218148 | 101219503 | 451 | Ⅱ-a |
| 16 | WvBAHD16 | Wv_051091 | LG12 | 3227340 | 3228818 | 492 | Ⅱ-a |
| 17 | WvBAHD17 | Wv_051113 | LG12 | 3379510 | 3380894 | 437 | Ⅰ-a |
| 18 | WvBAHD18 | Wv_054051 | LG13 | 6874415 | 6879744 | 442 | Ⅰ-b |
| 19 | WvBAHD19 | Wv_054053 | LG13 | 6902914 | 6933106 | 445 | Ⅰ-b |
| 20 | WvBAHD20 | Wv_054054 | LG13 | 6954840 | 6957380 | 439 | Ⅰ-b |
| 21 | WvBAHD21 | Wv_054121 | LG13 | 8579028 | 8580833 | 434 | Ⅰ-b |
| 22 | WvBAHD22 | Wv_054122 | LG13 | 8680995 | 8690797 | 465 | Ⅰ-b |
| 23 | WvBAHD23 | Wv_054125 | LG13 | 8704692 | 8719238 | 434 | Ⅰ-b |
| 24 | WvBAHD24 | Wv_054134 | LG13 | 8852369 | 8856724 | 434 | Ⅰ-b |
| 25 | WvBAHD25 | Wv_054135 | LG13 | 8864519 | 8885696 | 434 | Ⅰ-b |
| 26 | WvBAHD26 | Wv_054139 | LG13 | 8900745 | 8914900 | 361 | Ⅰ-b |
| 27 | WvBAHD27 | Wv_054141 | LG13 | 8964503 | 8985789 | 433 | Ⅰ-b |
| 28 | WvBAHD28 | Wv_054143 | LG13 | 8995186 | 9003985 | 435 | Ⅰ-b |
| 29 | WvBAHD29 | Wv_055100 | LG13 | 61103978 | 61105309 | 443 | Ⅱ-b |
| 30 | WvBAHD30 | Wv_055108 | LG13 | 61853107 | 61854441 | 444 | Ⅱ-b |
| 31 | WvBAHD31 | Wv_055627 | LG13 | 106392711 | 106394033 | 440 | Ⅰ-a |
| 32 | WvBAHD32 | Wv_055719 | LG13 | 108332180 | 108335517 | 459 | Ⅱ-b |
| 33 | WvBAHD33 | Wv_056269 | LG14 | 3942676 | 3944111 | 418 | Ⅰ-a |
| 34 | WvBAHD34 | Wv_056271 | LG14 | 4032806 | 4034308 | 418 | Ⅰ-a |
| 35 | WvBAHD35 | Wv_056274 | LG14 | 4066560 | 4087222 | 417 | Ⅰ-a |
| 36 | WvBAHD36 | Wv_060556 | LG16 | 4989030 | 4990391 | 453 | Ⅰ-a |
| 37 | WvBAHD37 | Wv_061097 | LG16 | 8715245 | 8720269 | 463 | Ⅰ-a |
| 38 | WvBAHD38 | Wv_063076 | LG17 | 26026981 | 26028321 | 446 | Ⅰ-a |
| 39 | WvBAHD39 | Wv_064582 | LG18 | 52321054 | 52322711 | 443 | Ⅰ-a |
| 40 | WvBAHD40 | Wv_064664 | LG18 | 54843462 | 54845786 | 427 | Ⅰ-a |
| 41 | WvBAHD41 | Wv_064667 | LG18 | 54988493 | 54990633 | 427 | Ⅰ-a |
| 42 | WvBAHD42 | Wv_064668 | LG18 | 55063951 | 55066326 | 429 | Ⅰ-a |
| 43 | WvBAHD43 | Wv_064682 | LG18 | 55438500 | 55446374 | 542 | Ⅰ-a |
| 44 | WvBAHD44 | Wv_065520 | LG19 | 4273856 | 4275280 | 432 | Ⅰ-a |
| 45 | WvBAHD45 | Wv_065982 | LG19 | 12172619 | 12173977 | 452 | Ⅱ-b |
| 46 | WvBAHD46 | Wv_066032 | LG19 | 13177629 | 13178963 | 444 | Ⅱ-b |
| 47 | WvBAHD47 | Wv_066044 | LG19 | 13384383 | 13388401 | 467 | Ⅱ-b |
| 48 | WvBAHD48 | Wv_070607 | LG21 | 82709104 | 82711142 | 539 | Ⅱ-b |
| 49 | WvBAHD49 | Wv_071258 | LG22 | 6642364 | 6644977 | 411 | Ⅰ-a |
| 50 | WvBAHD50 | Wv_001248 | Contig1062 | 7053103 | 7055391 | 419 | Ⅰ-a |
| 51 | WvBAHD51 | Wv_005355 | Contig326 | 95073 | 96543 | 455 | Ⅱ-a |
| 52 | WvBAHD52 | Wv_006346 | Contig339 | 4159547 | 4164024 | 469 | Ⅵ |
| 53 | WvBAHD53 | Wv_006347 | Contig339 | 4159289 | 4164024 | 461 | Ⅵ |
| 54 | WvBAHD54 | Wv_006548 | Contig356 | 474599 | 476267 | 466 | Ⅰ-b |
| 55 | WvBAHD55 | Wv_009144 | Contig49 | 903102 | 904385 | 427 | Ⅰ-b |
| 56 | WvBAHD56 | Wv_009952 | Contig554 | 1275553 | 1278318 | 521 | Ⅱ-b |
| 57 | WvBAHD57 | Wv_009953 | Contig554 | 1279717 | 1281054 | 445 | Ⅱ-b |
| 58 | WvBAHD58 | Wv_010491 | Contig554 | 5732032 | 5733488 | 436 | Ⅰ-a |
| 59 | WvBAHD59 | Wv_011311 | Contig594 | 841147 | 842550 | 467 | Ⅱ-b |
| 60 | WvBAHD60 | Wv_011719 | Contig594 | 4036705 | 4038526 | 426 | Ⅰ-a |
| 61 | WvBAHD61 | Wv_013915 | Contig709 | 2178475 | 2179836 | 453 | Ⅱ-a |
| 62 | WvBAHD62 | Wv_013916 | Contig709 | 2207592 | 2208953 | 453 | Ⅱ-a |
| 63 | WvBAHD63 | Wv_019974 | Contig948 | 256673 | 258091 | 472 | Ⅱ-a |
| 64 | WvBAHD64 | Wv_019980 | Contig948 | 311215 | 312660 | 481 | Ⅱ-a |

The BAHD clade nomenclature of clustering were adapted from Liu et al. (2020), which is consistent with the **Figure 3A**–**B**.

**Supplementary Table 5. Subcellular localization prediction of WvBAHDs**

| **No.** | **Gene name** | **SignalP** | **ChloroP** | **TMHMM** |
| --- | --- | --- | --- | --- |
| 1 | WvBAHD1 | N | N | N |
| 2 | WvBAHD2 | N | Y | N |
| 3 | WvBAHD3 | N | N | N |
| 4 | WvBAHD4 | N | N | N |
| 5 | WvBAHD5 | N | Y | N |
| 6 | WvBAHD6 | N | N | N |
| 7 | WvBAHD7 | N | N | N |
| 8 | WvBAHD8 | N | N | N |
| 9 | WvBAHD9 | N | N | N |
| 10 | WvBAHD10 | N | N | N |
| 11 | WvBAHD11 | N | N | N |
| 12 | WvBAHD12 | N | N | N |
| 13 | WvBAHD13 | N | N | N |
| 14 | WvBAHD14 | N | N | N |
| 15 | WvBAHD15 | N | N | N |
| 16 | WvBAHD16 | N | Y | N |
| 17 | WvBAHD17 | N | N | N |
| 18 | WvBAHD18 | N | N | N |
| 19 | WvBAHD19 | N | N | N |
| 20 | WvBAHD20 | N | N | N |
| 21 | WvBAHD21 | N | N | N |
| 22 | WvBAHD22 | N | N | N |
| 23 | WvBAHD23 | N | N | N |
| 24 | WvBAHD24 | N | N | N |
| 25 | WvBAHD25 | N | N | N |
| 26 | WvBAHD26 | N | N | N |
| 27 | WvBAHD27 | N | N | N |
| 28 | WvBAHD28 | N | N | N |
| 29 | WvBAHD29 | N | N | N |
| 30 | WvBAHD30 | N | N | N |
| 31 | WvBAHD31 | N | N | N |
| 32 | WvBAHD32 | N | Y | N |
| 33 | WvBAHD33 | N | N | N |
| 34 | WvBAHD34 | N | N | N |
| 35 | WvBAHD35 | N | N | N |
| 36 | WvBAHD36 | N | N | N |
| 37 | WvBAHD37 | N | Y | N |
| 38 | WvBAHD38 | N | N | N |
| 39 | WvBAHD39 | N | N | N |
| 40 | WvBAHD40 | N | N | N |
| 41 | WvBAHD41 | N | N | N |
| 42 | WvBAHD42 | N | N | N |
| 43 | WvBAHD43 | N | N | N |
| 44 | WvBAHD44 | N | N | N |
| 45 | WvBAHD45 | N | N | N |
| 46 | WvBAHD46 | N | N | N |
| 47 | WvBAHD47 | N | N | N |
| 48 | WvBAHD48 | N | N | N |
| 49 | WvBAHD49 | N | N | N |
| 50 | WvBAHD50 | N | N | N |
| 51 | WvBAHD51 | N | Y | N |
| 52 | WvBAHD52 | N | N | N |
| 53 | WvBAHD53 | N | N | N |
| 54 | WvBAHD54 | N | N | N |
| 55 | WvBAHD55 | N | N | N |
| 56 | WvBAHD56 | N | N | N |
| 57 | WvBAHD57 | N | N | N |
| 58 | WvBAHD58 | N | N | N |
| 59 | WvBAHD59 | N | Y | N |
| 60 | WvBAHD60 | N | N | N |
| 61 | WvBAHD61 | N | N | N |
| 62 | WvBAHD62 | N | N | N |
| 63 | WvBAHD63 | N | N | N |
| 64 | WvBAHD64 | N | N | N |

Y: the prediction result is positive; N: the prediction result is negative.

**Supplementary Table 6. Transcriptome differential expression of *WvBAHDs* belonging to clade Ⅰ and clade Ⅵ in different tissues.**

| **Clade** | **WvBAHD** | **Leaf** | **Rhizome** | **Flower** | **Pericarp-1** | **Pericarp-2** | **Seeds-1** | **Seeds-2** |
| --- | --- | --- | --- | --- | --- | --- | --- | --- |
| Clade  Ⅰ | WvBAHD1 | 0.01 | 11.87 | 0.45 | 11.78 | 25.74 | 22.30 | 9.98 |
|  | WvBAHD8* | 2.45 | 1.26 | 34.91 | 7.65 | 2.13 | 0.43 | 0.02 |
|  | WvBAHD9* | 9.06 | 54.03 | 30.77 | 49.09 | 16.98 | 82.58 | 46.11 |
|  | WvBAHD12 | 0.00 | 0.02 | 0.00 | 0.15 | 0.00 | 0.00 | 0.06 |
|  | WvBAHD13 | 0.01 | 1.42 | 2.36 | 10.91 | 5.86 | 17.94 | 0.60 |
|  | WvBAHD14* | 9.71 | 22.02 | 55.79 | 115.02 | 31.86 | 274.92 | 109.70 |
|  | WvBAHD17 | 15.04 | 4.02 | 4.11 | 9.97 | 9.35 | 1.46 | 2.80 |
|  | WvBAHD18* | 69.39 | 374.84 | 257.65 | 154.03 | 64.13 | 106.72 | 41.66 |
|  | WvBAHD19 | 0.00 | 1.01 | 0.80 | 0.75 | 0.15 | 5.36 | 0.26 |
|  | WvBAHD20 | 0.00 | 0.02 | 0.22 | 0.02 | 0.00 | 0.12 | 0.00 |
|  | WvBAHD21 | 28.92 | 0.00 | 0.01 | 0.04 | 0.00 | 0.09 | 0.00 |
|  | WvBAHD22 | 4.57 | 0.00 | 0.00 | 0.03 | 0.01 | 0.02 | 0.03 |
|  | WvBAHD23 | 2.67 | 8.32 | 0.78 | 16.19 | 16.57 | 5.95 | 1.82 |
|  | WvBAHD24 | 0.00 | 5.25 | 0.01 | 0.82 | 3.85 | 0.00 | 0.04 |
|  | WvBAHD25 | 0.61 | 99.06 | 0.70 | 37.76 | 71.75 | 0.96 | 0.52 |
|  | WvBAHD26 | 2.08 | 1.02 | 0.04 | 0.00 | 0.00 | 0.00 | 0.00 |
|  | WvBAHD27 | 1.33 | 0.00 | 0.18 | 0.01 | 0.00 | 0.04 | 0.00 |
|  | WvBAHD28* | 0.11 | 0.00 | 1.01 | 23.66 | 2.68 | 237.18 | 272.91 |
|  | WvBAHD31* | 7.53 | 18.49 | 2.94 | 20.90 | 23.30 | 18.21 | 19.59 |
|  | WvBAHD33 | 0.62 | 4.52 | 2.06 | 0.45 | 0.10 | 0.65 | 8.25 |
|  | WvBAHD34 | 1.96 | 12.67 | 1.66 | 3.25 | 1.25 | 0.01 | 0.02 |
|  | WvBAHD35 | 3.51 | 48.12 | 4.29 | 6.24 | 3.36 | 0.05 | 0.02 |
|  | WvBAHD36 | 0.04 | 0.00 | 2.83 | 0.00 | 0.00 | 0.10 | 0.00 |
|  | WvBAHD37 | 0.10 | 0.54 | 0.00 | 0.00 | 0.00 | 0.01 | 0.00 |
|  | WvBAHD38* | 10.35 | 29.17 | 0.31 | 1.93 | 4.61 | 0.95 | 1.42 |
|  | WvBAHD39* | 0.04 | 0.42 | 0.21 | 0.17 | 0.86 | 0.03 | 71.10 |
|  | WvBAHD40* | 0.00 | 0.00 | 0.01 | 0.00 | 0.30 | 0.00 | 57.11 |
|  | WvBAHD41 | 3.94 | 0.06 | 1.68 | 0.23 | 0.38 | 0.00 | 1.47 |
|  | WvBAHD42 | 0.00 | 0.00 | 0.27 | 0.00 | 0.00 | 0.05 | 0.00 |
|  | WvBAHD43 | 0.00 | 2.80 | 0.18 | 0.07 | 0.01 | 0.24 | 0.04 |
|  | WvBAHD44 | 2.39 | 6.95 | 0.13 | 1.34 | 3.58 | 0.10 | 2.44 |
|  | WvBAHD49 | 6.72 | 23.31 | 4.70 | 23.35 | 11.62 | 11.38 | 2.41 |
|  | WvBAHD50* | 0.18 | 18.80 | 5.97 | 164.61 | 35.00 | 473.66 | 323.86 |
|  | WvBAHD54 | 0.00 | 4.20 | 0.00 | 23.17 | 82.02 | 19.60 | 91.32 |
|  | WvBAHD55 | 0.04 | 0.14 | 0.10 | 0.05 | 0.16 | 0.09 | 2.30 |
|  | WvBAHD58 | 0.00 | 0.41 | 0.09 | 28.76 | 0.77 | 303.22 | 0.29 |
|  | WvBAHD60 | 31.23 | 26.93 | 12.97 | 38.78 | 8.91 | 6.17 | 1.65 |
| Clade Ⅵ | WvBAHD3* | 155.62 | 502.62 | 74.69 | 48.95 | 76.52 | 0.42 | 23.26 |
|  | WvBAHD52 | 0.24 | 0.00 | 0.00 | 0.39 | 0.00 | 0.00 | 0.07 |
|  | WvBAHD53* | 64.40 | 24.58 | 38.57 | 174.12 | 92.71 | 89.84 | 94.48 |
| - | WvBPPS | 0.01 | 0.00 | 3.03 | 0.03 | 17.15 | 0.28 | 3543.35 |

1: the developmental stage of 30-DAF (DAF: days after flowering); 2: the strage of 60-DAF. The genes with asterisk are the WvAAT candidate genes.

**Supplementary Table 7. The deduced amino acid sequences of eight WvBATs**

| **WvBAT** | **Amino acid sequence** |
| --- | --- |
| WvBAT1  (WvBAHD8) | MASSSLTFTVRRRDAVLVAPAEATPREFKRLSDVDDQDGLRFHIPVIQFYGSHPSMAGQDPCKVIREALARALVFYYPFAGRLRETEGRKLVVECTGEGVLFIEADADVRLDQFGDALQPPFPGLEELLWNVPGSDGVLHCPLLLIQVTRLLCGGFVFALRLNHTMCDAPGLVQFMNAVAELARGAAAPSLAPVWSREVLEARHPPQVTCVHREYEDVPGTIVPFADMVHRSFFFGKAEVAALRRRVPKHLRNSSTFEILTACLWKCRTVAIGANSDEEVRIICIVNARGKSGLGLPPGYYGNAFAFPVAVSTAGKLSGNPIGYALDLVKKAKSAVSDEYMRSLSDLMVLRGRPHFTVVRSYLVSDVTRAGFGDVDFGWGKAAYGGPAKGGVGAIPGVASFYIPLKNSKGEEGIVVPVCLPASAMDKFTQEMHNLVKEDGDKDAKERQPQGVLIVSAL |
| WvBAT2  (WvBAHD14) | MGFTVTKTSEGYVRPAEPTPPGSLTLDWIGRYPTHRGLVDSLHIYKHGQNPAEVIREALARALVPYYPIAGRIVQPEGCEPRIECTGEGVWFVAASANCSLADVNYLERPMMLGQDDILPYTELETSSANTLMMIQVTEFTCGGFLIGLRFNHASADGLGSAQVISAIGDLARGLPEPTIKPVWNRDSYPNPKIKPAPLPDLPKLALEYSAVDFPAHYIDQLKKQYMEHSKGKWCSTFDIIIAKVWQCRTRAIYSDPDVNIRMCFFASTRHILKIEKGYYGNSIFPVKVSTTSGKVNNLSVVEIVDLIKEAKDQMAIDVLKWANDEFEVDPFSMTFNYETIYVSDWTKLGFSGVDYGWGTPMYCGPFTNNDYIASCILLKAPAPFEGARLIARCVSKEHIDAFNERVNLFD |
| WvBAT3  (WvBAHD39) | MSMASWVTKVAEELVAPCKATPCATLPLSSIDHALNLAFMQEMISVYPNNNRQHRRHDVSLPAAKVIREALSKALVPYYPVAGRLVSSGPGDRVEVACNGEGVWFVEAAVINRSLGDINDWESIPRSVLKEELVPNCPAHLNQEEMMLMMQVTDFQCGGFIVGLKFNHLVFDGIGIGQFLKAIGEITCGQTHPSINPIWDRETIPNPPMLSKSLPLLPAKLDLVNSMYDFSIQTVKRLKEKIAKETSNQFTTFELMAAIIWKCRTQAISVVGDVGLTFAANIRHLLCQLPKAGYYGNCVCALTITATSEQIMKASLAELVGIIRDAKESLPTKFKEWSSGNFNEDPFKVSTSYNNLILSDWRWMEFYETDYGWGIPHSISPRTHDFSLFTCGIILKQPFPKDGVRFEGQLVMKEHEQRFIDEINKCINEEKVSHNGQIDFPLE |
| WvBAT4  (WvBAHD40) | MSMASWVTKVAAEELVAPCEPTPCATLPLSSIDHALGLAFMVEMISIYPNNNRQHHHPAAKVIQEALAKALVPYYPVAGRLVSSGGDCVEVACNGEGVWFVEAMVTDHSLNGLNDWESIPRSVVKDELIPGCPSHLKQEEMIMMMQVTHFQCGGFIVGLKFNHLVFDGLGFGQFLKAIGEIACGRAHPSVDPIWYREAIPVPFMLSKSISFPITKIDIVNSSYDFSIQTIKRLKGQIANETSNQFTTFEVVVAILWKCRTQAINAIGDVCLSFPANVRYLLDQLPKAGGYYGNCIYNLTVTATSEQIKKASLAELVRLIRNAKESLPTKFKEWTSGNFKEDPYKISSSYNSLILSDWRCIAIDEADFGWGLPHFVSPIMHDIPFASGIILKQSLPKGGVHFEGLATMKEHEKRFIDEINKFLANVML |
| WvBAT5  (WvBAHD50) | MSFSVTRTSQSFVAPASPTPAETLFLSIIDRVAGLRHMVRSIHVFKDGRESAKVIREALAKALVPYHPFAGRFVDDAEHGDVRVACTGEGTWFVEATANCSLEDVRDLDFPLMISKDELLPVPSHEFDPINVPLMMQVTEFTCGGFVVGLISVHTIADGLGAAQFVNAIGDIARGLPKPAVDPVWSREVIPSPPKLAPSAPPLFDSFKLVHTTMDIPESAVNQMKAKYLEHTGQRCSTFDIAIAKLWQSRTRAIGLSDDADVHLYFFANTRHLMQQVLPRGYFGNCFYPVSITACSGEVAGAELVEVVRMIRDAKAGLAAEFGRWATGDFKADPYELTFSYNSLFVSDWTRLGFLDVDYGWGKPLHVIPFAYFDFMAVGIIGAPPLPKTGTRIMTQCVEKEQLETFMEEMSSSDGVHAA |
| WvBAT6  (WvBAHD28) | MVVINVRRSTLVRPAETTPQRRLWLSNLDLVTERGHSPIVYFYRPDGSANFFDAVVLRDALARVLVPFYPMAGRLARGEDGRIAIDCNGEGVLFVEADTEATVDDFGDFAPTGELEQLIPVLNADYADGISAFPLLTLQVTHLKCGGAALGTGMHHWTADGFAAIHMINSWSDVARGIGITVQPFLDRTLVRGRDPPNPSFAHVEYQLPFPSLSSSAARVLPSSAAVVRVFKFTREQLNLLKAKAPPDGGYSTHVVLAASVWRCACIACDFPPDQVTRMYIATNGRQRIQPPLPQGYFGNAIFMAAAIAAVGEVASPEGGAYSAAKTIQEAVLRMDAAYLQSALDYLEMHPNLEAPVRGGAPLRCSMLGFVSWARLPIHDADFGWGRPIFMGPARIDFEGLTFVLPSAAGDGGLSVVISLQPDHMVKFQELIYDI |
| WvBAT7  (WvBAHD3) | MASGEAPSRALGGTENGWWRAMPGGTGVSVLIFLPTRPISRPLLESAVRSLQSSHPILRSFLSAPTPGQPTLTVASAPSATVRVVAASDVLPAEPGPVSSFHALLERELNQNAWAEQGPADPILYATIYEGVPEEGRSAVALRMHSSACDRVSVGRVLKELVQLLAGGEKGTKEEPFHAALEDLIPKADTYKPFWARGKDMIGYGINGLRTSPLPFEDTVTGRRSEVARLVLSRDETQKLLSECKERGAKLCGAIGAAAMIAARSSKQLESHQYETYSLVTLIDCRKNLDPPLNGQNLGNFQSAVINTHNIQGEENFWELVGRCHESYHTAVTNKKHLKDINDLNFLFLRAIDNPQLTPSASQRTALVTVFEESAIHESSELPEELGVEDYIGCSSVHGIGPSLAVFDTIRDGKLDCAIVYPAPLHSRKQIGDLLDHIKRILLGSEEE |
| WvBAT8  (WvBAHD53) | MSEADAAGRPVGGTEDSWIRAVPGGTGTTVLALLLSRPVCLTLLHSALRRLQASHRLLFAHLTTASTAHHFFSLTDPYSISILSLNASDLPPPPPAAEAGISSFHAILEREINQNPWSDPCPDRLPLLFATVYEMPDPARTVLALRFHTAVCDRTAAVAVLKELLCLMSSDGDGGLAEGLNREIEELIPRQDAWKPFWARGKDLLGYSLNALRTSTLRFEDASCHVRRSEVARLMLGADATEKLLTECKARAIKLCGAISAAAMLATHASKKFENDQYETYSVVTLIDCRKYLDPVLHDHNMGFYHSAIINTHSIYSGEGLWEVAKRCQDSYSNAMNNKKHLKDIGELNFLMCRAIENPQLTPSSSLRTALISVFEEPVVHDSSKLQEEVGLDDYFGCASVHGVGPSIAVFDTIRDGKLDCACVYPSPLHSRNQIQELLEQMVRLLTQGTLNGESKEIEKY |

| **Supplementary Table 8. The information of identified BAHD members** | | |
| --- | --- | --- |
| **BAHD member** | **Definition** | **Accession** **number** |
| AcAT16 | *Actinidia chinensis* alcohol acyltransferase | HO772640 |
| AeAT9 | *Actinidia deliciosa* alcohol acyltransferase | HO772637 |
| ApAAT | *Malus sp.*（Apple-） alcohol acyltransferase | CAC09064 |
| AsHHT1 | *Avena sativa* hydroxycinnamoyl-CoA: hydroxyanthranilate-N-hydroxycinnamoyltransferse | BAC78633 |
| AtAT | *Arabidopsis thaliana* acyltransferase | NP_197782 |
| AtCER2 | *A. thaliana* CER2-protein, biosynthesis of C30-waxes | AAM64817 |
| AtCHAT | *A. thaliana* (Z)- 3-hexen-1-ol-O-acetyltransferase | AAN09797 |
| AtHCT | *A. thaliana* hydroxycinnamoyl-CoA: shikimate/quinate hydroxycinnamoyltransferase | NP_199704 |
| CaAT20 | *Celastrus angulatus* BAHD acyltransferase | QLI57449 |
| CaPun1 | *Capsicum annum* Pun1-Protein, biosynthesis of capsaicin | AAV66311 |
| CbBEAT | *Clarkia breweri* benzyl alcohol-O-acetyltransferase | AAC18062 |
| CbBEBT | *C. breweri* benzoyl-CoA: benzyl alcohol-O-benzoyltransferase | AAN09796 |
| CmAAT  1~4 | *Cucumis melo* alcohol acyltransferase | CAA94432  AAl77060 AAW51125 AAW51126 |
| CmMAAT | *Cucumis melo* (muskmelon-/Honey dew melon-) alcohol acyltransferase | CAC09068 |
| CrDAT | *Catharanthus roseus* deacetylvindolin-4-O-acetyltransferase | AAC99311 |
| CrMAT | *C. roseus* minovincinin-19-hydroxy-O-acetyltransferase | AAO13736 |
| DcHCBT | *Dianthus* caryophyllus anthranilate-N-hydroxycinnamoyl/ benzoyltransferase | CAB06430 |
| DkAAT | *Diospyros kaki* alcohol acyltransferase 1 | AKE98481 |
| Dm3MAT  1~2 | *Dendramthema x morifolium* anthocyanidine-3-O-glucosid-6''-O-malonyl-transferase | AAQ63615 AAQ63616 |
| Dv3MAT | *Dahlia variabilis* malonyl-CoA: anthocyanidine-3-O-glucosid-6''-O-malonyltransferase | AAO12206 |
| EiAAT | *Eriobotrya japonica* alcohol acyltransferase 1 | AHC32224 |
| FaSAAT | *Fragaria x ananassa* (strawberry-) alcohol acyltransferase | AAG13130 |
| FaAAT2 | *F. x ananassa* (strawberry-) alcohol acyltransferase | AEM43830 |
| FvVAAT | *Fragaria vesca* alcohol acyltransferase | CAC09062 |
| GsAAT | *Camellia sinensis* alcohol acyltransferase | ACV74416 |
| Gt5AT | *Gentiana triflora* anthocyanin-5-aromatic acyltransferase | BAA74428 |
| LaAT  1~2 | *L. angustifolia* acyltransferase 1 and 2; | ABI48360 ABI48361 |
| ClLAAT  1-4 | *Citrus limon* (Lemon-) acyltransferase | CAC09049  CAC09067  CAC09066  CAC09065 |
| LiAAT-  3~4 | *Lavandula x intermedia* alcohol acetyltransferase | AIW81432 AIW81431 |
| Lp3MAT1 | *Lamium purpureum* malonyl-CoA: flavonol-3-O-glucosid- 6''-O-malonyl- transferase | AAS77404 |
| LuaHMT  /  HLT | *Lupinus albus* tigloyl-CoA: 13a-hydroxymultiflorin/13a-hydroxy-lupanin- O-tigloyltransferase | BAD89275 |
| MdAAT | *Malus domestica* alcohol acyltransferase | AAR99826 |
| MdAAT1 | *M. domestica* alcohol acyltransferase | AGW30203 |
| MdAAT2 | *M. domestica* alcohol acyltransferase | AAS79797 |
| MdAAT3 | *M. domestica* truncated alcohol acyltransferase | AGW30204 |
| MpAAT1 | *Malus pumila* (pumila-) alcohol acyltransferase | AAU14879 |
| MsBanAAT | *Musa sapientum* (banana-) alcohol acyltransferase | CAC09063 |
| NtBEBT | *Nicotiana tabacum* benzoyl-CoA: benzyl alcohol-O-benzoyltransferase | AAN09798 |
| NtHCT | *N. tabacum* hydroxycinnamoyl-CoA: shikimate/ quinate hydroxycinnamoyltransferase | CAD47830 |
| NtHQT | *N. tabacum* hydroxycinnamoyl-CoA: quinate hydroxycinnamoyltransferas | CAE46932 |
| NtMAT | *N. tabacum* malonyl-CoA: flavonoid/naphthol-glucosid-acyltransferase | BAD93691 |
| OsAT10 | *Oryza sativa Japonica* acyltransferase 10 | LOC_  Os06g39390 |
| OsAT5 | *Oryza sativa Japonica* acyltransferase 5 | LOC_  Os05g19910 |
| PaAAT1 | *Prunus armeniaca* alcohol acyltransferase | ACF07921 |
| PcAAT | *Pyrus communis* alcohol acyltransferase | AAS48090 |
| Pf5MAT | *Perilla. frutescens* anthocyanin-5-O-glucosid-6''-O-malonyltransferase | AAL50565 |
| PhAT | *Petunia hybrida* acyltransferase | BAA93453 |
| PhBPBT | *P. hybrida* benzoyl-CoA: benzyl alcohol/phenylethanol-benzoyltransferase | AAU06226 |
| PpAAT1 | *Prunus persica* alcohol acyltransferase | DY645545 |
| PsSaIAT | *Papaver somniferum* salutaridinol-7- O-acetyltransferase | AAK73661 |
| PuAAT | *Pyrus ussuriensis* alcohol acyltransferase | AJD18611 |
| RhAAT1 | *Rosa hybrida* alcohol acetyltransferase | AAW31948 |
| RsVinS | *Rauwolfia serpentina* vinorinsynthase | CAD89104 |
| Sc3MaT | *Senecia cruentus* malonyl-CoA: anthocyanidin-3-O-glucosid-6''-O-malonyl-transferase | AAO38058 |
| SlAAT1 | *Solanum lycopersicum* alcohol acyltransferase | KM975322 |
| SpAAT1 | *Solanum pennellii* alcohol acyltransferase | KM975321 |
| SlTomAAT | *Solanum lycopersicum/Lycopersicon esculentum* (tomato-) alcohol acyltransferase | AAS48091 |
| Ss5MAT1 | *Salvia splendens* malonyl-CoA; anthocyanin-5-O-glucosid-6''-O-malonyltransferase | AAL50566 |
| Ss5MAT2 | *S. splendens* anthocyanin 5-O-glucoside-4'''-O-malonyltransferase | AAR26385 |
| SsRAS | *Solenostemon scutellarioides* rosmarinic acid synthase | CAK55166 |
| TcaDBTNBT | *Taxus Canadensis* 3’-N-debenzoyltaxol-N-benzoyltransferase | AAM75818 |
| TcBAPT | *Taxus cuspidata* baccatin-III-O-phenylpropanoyltransferase | AAL92459 |
| TcDBAT | *T. cuspidata* 10-deacetylbaccatin-III-10-O-acetyltransferase | AAF27621 |
| TcDBBT | *T. cuspidata* 2-debenzoyl-7,13-diacetylbaccatin-III-O-benzoyltransferase | Q9FPW3 |
| TcTAT | *T. cuspidata* taxa-4(20),11(12)-dien-5a-ol-O-acetyltransferase | AAF34254 |
| Vh3MAT1 | *Verbena x hybrida* malonyl-CoA: flavonol-3-O-glucosid-6''-O-malonyltransferase | AAS77404 |
| VlAMAT | *Vitis labrusca* anthranoyl-CoA:methanolacyltransferase | AAW22989 |
| VpAAT | *Vasconcellea cundinamarcensis* (mountain papaya) alcohol acyltransferase | ACT82248 |
| VvAAT1 | *Vitis x labruscana* alcohol acyltransferase | ART85743 |
| ZmGlossy2 | *Zea mays* Glossy2-protein, biosynthesis of C32-waxes | CAA61258 |
